# Human leukocyte immunoglobulin-like receptors in health and disease

**DOI:** 10.3389/fimmu.2023.1282874

**Published:** 2023-11-13

**Authors:** Silvia Redondo-García, Christopher Barritt, Charys Papagregoriou, Muchaala Yeboah, Björn Frendeus, Mark S. Cragg, Ali Roghanian

**Affiliations:** ^1^ Antibody and Vaccine Group, Centre for Cancer Immunology, School of Cancer Sciences, Faculty of Medicine, University of Southampton, Southampton General Hospital, Southampton, United Kingdom; ^2^ Lister Department of General Surgery, Glasgow Royal Infirmary, Glasgow, United Kingdom; ^3^ School of Medicine, Dentistry and Nursing, University of Glasgow, Glasgow, United Kingdom; ^4^ BioInvent International AB, Lund, Sweden; ^5^ Institute for Life Sciences, University of Southampton, Southampton, United Kingdom

**Keywords:** LILR, immune tolerance, cancer, autoimmunity, infection, immunomodulation, immunotherapy

## Abstract

Human leukocyte immunoglobulin (Ig)-like receptors (LILR) are a family of 11 innate immunomodulatory receptors, primarily expressed on lymphoid and myeloid cells. LILRs are either activating (LILRA) or inhibitory (LILRB) depending on their associated signalling domains (D). With the exception of the soluble LILRA3, LILRAs mediate immune activation, while LILRB1-5 primarily inhibit immune responses and mediate tolerance. Abnormal expression and function of LILRs is associated with a range of pathologies, including immune insufficiency (infection and malignancy) and overt immune responses (autoimmunity and alloresponses), suggesting LILRs may be excellent candidates for targeted immunotherapies. This review will discuss the biology and clinical relevance of this extensive family of immune receptors and will summarise the recent developments in targeting LILRs in disease settings, such as cancer, with an update on the clinical trials investigating the therapeutic targeting of these receptors.

## 1 Introduction

The human immune system is composed of a network of complex effector cells, organs and tissues, all of which are tightly regulated to maintain immune homeostasis (1). One axis of immune regulation is through the dynamic integration of signals from the myriad of leukocyte activating and inhibitory cell surface receptors (1, 2).

Inhibitory receptors have recently been in the spotlight due to the development of immune checkpoint inhibitors for cancer immunotherapy. Current immunotherapies directed against the inhibitory receptors, such as programmed cell death protein 1 (PD-1) and cytotoxic T-lymphocyte-associated protein 4 (CTLA-4), have shown efficacy in various types of cancers that were previously untreatable (3). In addition, CTLA-4 Ig (abatacept) is being used to treat a number of autoimmune conditions, such as rheumatoid arthritis (RA) and type 1 diabetes (4). A variety of other cell-surface receptors are implicated in the regulation of the immune system and are potential targets for immunotherapy. One such family of receptors are the human LILRs, which play key roles in a wide range of immunological processes. Their ligation through interaction with endogenous or exogenous ligands can reprogram leukocytes and alter their functions (5, 6). Given their central roles in immunoregulation, LILRs are implicated in several pathologies. Hence, their targeting provides an attractive approach for the treatment of human disease.

This review will discuss LILR biology, immune responses mediated by each LILR, and their contribution to human health and disease. Furthermore, it will discuss the potential of targeting LILRs in treating a broad-spectrum of disorders, ranging from cancer to autoimmunity with reference to ongoing clinical trials.

## 2 LILR family

LILRs are a family of immune receptors with immunomodulatory roles in innate and adaptive immunity. The LILR gene family were independently discovered by different investigators around the same time (7). LILRs were originally identified in 1997 by the Colonna laboratory (8), followed by the Cosman laboratory (9). Due to their discovery by different investigators, these genes were assigned several different names (*e.g.*, ILT, LIR, MIR, CD85). LILR is the current standardised nomenclature for this receptor family, which was approved by the HUGO gene nomenclature committee in 2015 (10). LILRs are classified into two subfamilies: activating (LILRA) and inhibitory (LILRB).

### 2.1 Genetics, expression and structure of LILRs

LILRs are type 1 transmembrane glycoproteins structurally and functionally similar to killer cell Ig-like receptors (KIR) expressed on natural killer (NK) cells and some subsets of T lymphocytes (11, 12). *LILR* genes are located adjacent to the *KIRs* within the leukocyte receptor complex on chromosome 19 at 19q13.4, encoding for 11 functional genes and two pseudogenes (13, 14). The *LILR* gene cluster is believed to have originated from an activating founder gene, which after gene duplications gave rise to the current family and organisation (12). The *LILR* region consists of around 497 kb, divided into telomeric (~211 kb) and centromeric (~154 kb) regions, separated by a central region (~132 kb) (12). There are multiple polymorphisms in the receptor binding site of the LILRs (13, 15–20). In particular, *LILRB3* and *LILRA6* are considered as highly polymorphic and are found as different allelic variants, while *LILRA3* and *LILRA6* show copy number variations (13, 15–20). Interestingly, *LILRA3* shows an extremely high allele frequency of deletion in the Japanese population (21). In addition, although the *LILR* region in humans is relatively stable, a haplotype lacking *LILRA3* due to a 6.7 kb deletion exists (12).

**Figure 1:**
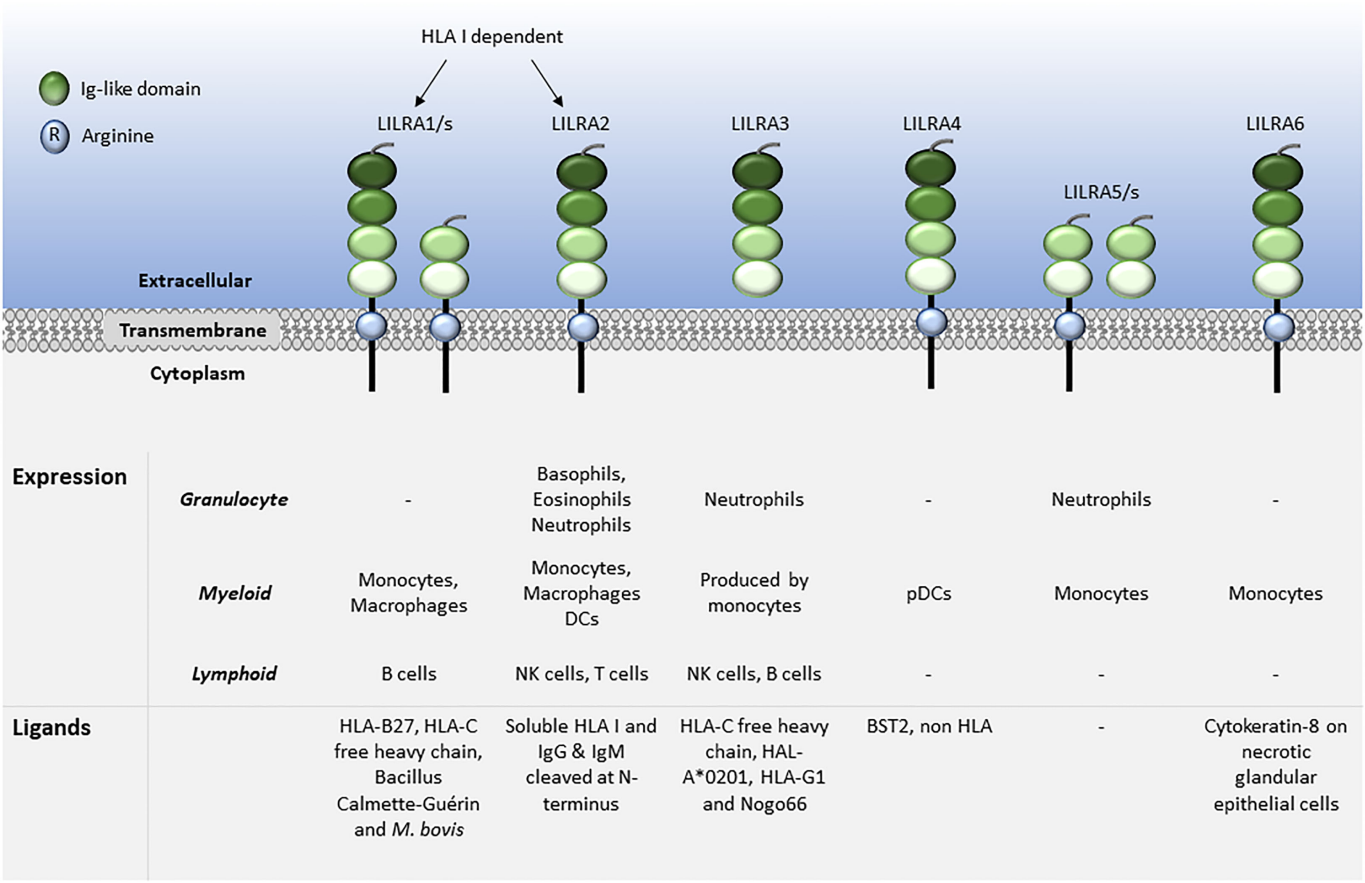
Leukocyte expression and ligand profiles of LILRAs. LILRAs have 2 to 4 extracellular lg-like domains, a transmembrane domain with a positively charged arginine residue and a truncated intracellular tail.

**Figure 2:**
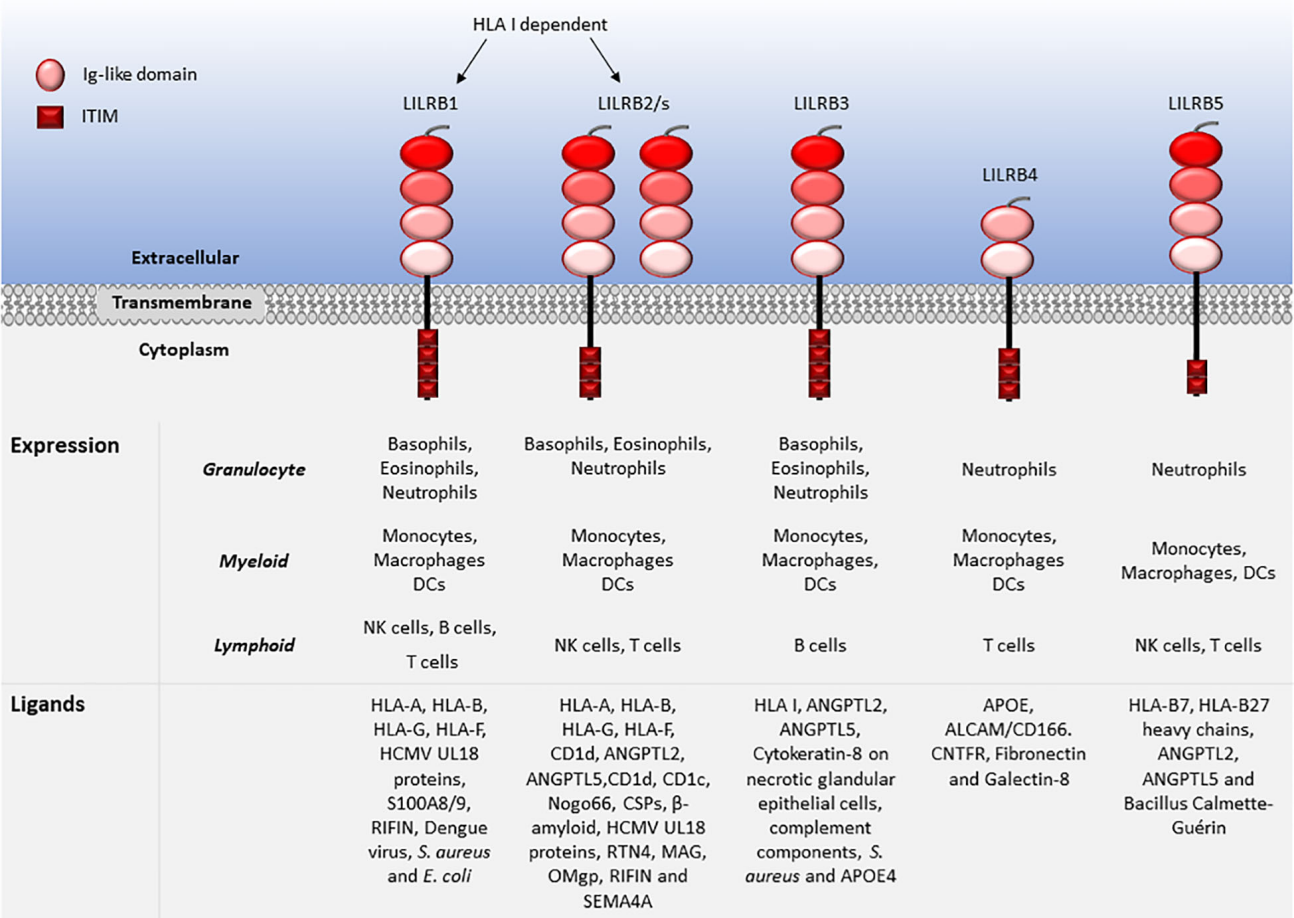
Leukocyte expression and ligand profiles of LILRBs. LILRBs have 2 to 4 extracellular lg-like domains and their cytoplasmic regions are composed of long ITIM-containing motifs exhibiting their inhibitory nature.

LILRs are primarily expressed on myeloid antigen-presenting cells (APC), such as monocytes and dendritic cells (DC), but also on granulocytes, NK cells, T and B lymphocytes, hematopoietic stem cells (22, 23), and non-immune cells, such as endothelial cells and neurons (12, 19) (**Figures 1**, **2**). LILRs are membrane-bound receptors, except for LILRA3. However, all LILRs also exist in soluble form as a result of alternative splicing (11, 19, 24). In addition, extracellular Ig-like domains of LILRB1, LILRB2, LILRB4, LILRA1, LILRA3 and LILRA5 are found in human sera or the supernatants of leukocytes (11, 25–29). These soluble LILR variants may act as decoy receptors, as demonstrated for LILRB1 (26).

**Figure 3:**
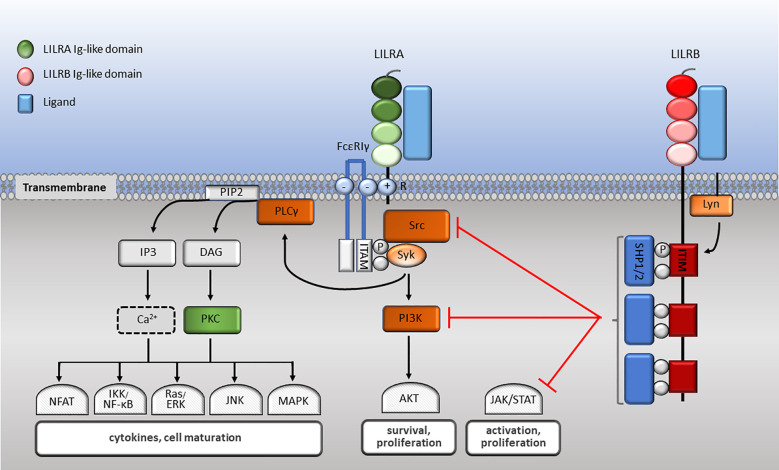
LILR signalling pathways. LILRA intracellular domain interacts with the dimeric FcεRIγ-chain comprised of cytoplasmic ITAM motifs. Phosphorylation of ITAM-bearing tyrosine residues by Src family kinases recruits Syk that mediates activating signalling cascades. Upon LILRB ligation, activated Lyn phosphorylates their ITIM domains, engaging phosphatases, which in turn abrogate activating signalling cascades essential for effector cell maturation and function.

The family have 2-4 C2-type Ig-like domains in their extracellular domains. However, their different cytoplasmic tails transduce either activating or inhibitory signalling (14). Apart from LILRA3 that only exists as a soluble form, the other five activating receptors (LILRA1, LILRA2, LILRA4, LILRA5 and LILRA6) have a shorter cytoplasmic tail and a positively charged arginine residue in the transmembrane domain (**Figure 1**). As a result, LILRAs transduce signals through an association with immunoreceptor tyrosine-based activation motif (ITAM)-containing high affinity IgE Fc epsilon receptor type I γ chain (FcεRIγ) (30) (**Figure 3**). In contrast, the five inhibitory receptors (LILRB1-5) signal through their immunoreceptor tyrosine-based inhibitory motifs (ITIM) (14, 30) (**Figure 2**). Together, LILRs fine-tune the immune response according to relevant local stimuli. Their dysfunction is therefore associated with pathologies ranging from autoimmunity to immunosuppression.

The crystal structure of a number of LILRs have been resolved. A partial structure of LILRB1 (distal D1 and D2) was resolved at 2.1Å resolution, confirming an Ig-like structure for both extracellular domains. It comprises β-sheets with fused helical regions, with a similar LILRB1 folding arrangement to that of the homologous KIR molecules (31). Nam et al. resolved the two membrane-proximal domains (D3 and D4) of LILRB1, as structurally similar to D1 and D2 (32). Based on the LILRB1 crystal structure model, Willcox et al. resolved LILRB2 by homology modelling to 1.8Å resolution (33). The folding of LILRB2 was predicted to be similar to LILRB1 apart from fewer α-helical structures (33). At the ligand binding interface, limited plasticity and flexibility were reported for both receptors due to the angles between the domains and the staggered assembly of the Ig-like domains (34). Willcox and colleagues further reported the crystal structure of the extracellular D1 and D2 of LILRB1 (35). The LILRB4 ectodomain was resolved at 1.7Å, depicting two Ig-like domains, similar in structure to the other LILR members. Although, LILRB4 D2 is similar to D4 of other LILRs, it contains helices that have not been reported before for this family. Reduced interdomain contact sites were also observed at the D1-D2 interface, which was associated with an obtuse interdomain angle of 107° (36). The crystal structure of LILRA2 indicated shifts in the amino acid residues that determine binding to human leukocyte antigen (HLA), explaining why it does not bind to HLA (37). The crystal structure of LILRA5 has also been reported (38), but the structures of other LILRs have not yet been determined.

The putative murine orthologues of LILRs are the paired Ig-like receptors (PIR), which possess six Ig-like domains and, similar to LILRs, are activating (PIR-A) or inhibitory (PIR-B) (19, 39, 40). PIR-B is the human ortholog of LILRB2-3, while glycoprotein 49B1 (gp49B1) is an orthologue of LILRB4 (41). Similar to *LILRs*, *PIR* genes are located within the leukocyte receptor complex on chromosome 7 (39). Resembling its human counterparts, PIR-A associates non-covalently with the ITAM-bearing FcγR adaptor molecule to transduce signals (42), while PIR-B contains 4 ITIMs in its cytoplasmic tail and binds to mouse major histocompatibility complex class I (39). These paired receptors are expressed on B cells, DCs, monocytes, macrophages, neutrophils, eosinophils, mast cells and megakaryocytes (42–47). These similarities in genomic location, expression profiles, structure and ligand affinity have identified PIR-A/B as the murine orthologues of human LILRs (19, 39, 40, 42). However, PIRs exhibit low overall homology to human LILRBs ranging from 45% to 54% as well as a wider tissue expression and greater regulatory effects than LILRs. Consequently, knowledge of PIRs (and gp49B1) may be limited when extrapolating to LILR biology.

### 2.2 LILR ligands

LILRs were initially characterised as HLA class I (HLA I) binding molecules. Later studies demonstrated that LILRs can be classed into two groups based on their ligands. Group 1 LILRs (LILRA1, LILRA2, LILRA3, LILRB1 and LILRB2) contain highly conserved HLA I binding sites, enabling the interaction with classical and non-classical HLA I or HLA I-like proteins. In contrast, group 2 LILRs (LILRA4, LILRA5, LILRA6, LILRB3, LILRB4 and LILRB5) interact with HLA I/β2-microglobulin (β2m) independent ligands (35). In this second group, LILRB5 is an exception since it interacts with angiopoietin-like proteins (ANGPTL) but also binds to HLA I heavy chains (12). Ligand profiles and known immunoregulatory functions of LILRs are summarised in **Table 1** and **Figures 1**, **2**.

**Table 1:** Overview of the expression, ligands and physiological functions of LILRs in health and disease.

Receptor	Other Names	Expression	Ligands	Physiological Functions	Human Cancers	Other conditions
***LILRA1***	LIR-6, CD85i	Monocytes, macrophages, B cell and mast cell progenitors (11, 12)	HLA-B27 (48), HLA-C free heavy chain (29) Bacillus Calmette-Guérin and *Mycobacterium bovis* (49)	–	–	HIV (50, 51) Allotransplantation (52)
***LILRA2***	LIR-7, ILT1, CD85h	Monocytes, macrophages, DCs, NK cells (30), basophils (53), eosinophils (54), neutrophils (55), T cells (14, 56), and mast cell progenitors (12)	Soluble HLA I (57) Microbially-cleaved IgG and IgM at N-terminus (55)	Ca^+^ influx in monocytes, pro-inflammatory cytokines release and degranulation of granulocytes (54, 58) Inhibition of monocyte differentiation to DCs and Ag presentation (59) Neutrophil and monocyte activation (55)	Oestrogen receptor^+^ breast cancer (60)	Leprosy (59, 61), bacterial infection (55), Salmonellosis (56) and HIV (50, 51) SLE, microscopic polyangiitis (62) and RA (63)
***LILRA3***	LIR-4, ILT6, CD85e, HM43, HM31	Monocytes, secreted in soluble form only (9, 15, 30), NK cells, B cells (14) and neutrophils (12)	HLA-C free heavy chain (29), HLA-A*0201, HLA-G1 (64) Nogo66 (65)	Release of pro-inflammatory cytokines and indirect activation of T and NK cells (66)	Non-Hodgkin lymphoma (66) and prostate cancer (67)	SS (68–70), SLE (69, 71), RA (25, 72, 73), MS (68, 74–76), ankylosing spondylitis, intestinal bowel disease (12) and AOSD (77)
***LILRA4***	ILT7, CD85g	Plasmacytoid DCs (78)	BST2 (79) Non HLA (80)	Inhibition of pDCs (Ca^+^ influx and IFN production) (80, 81)	PDA (82)	CLE (83–85), COVID-19 (86)
***LILRA5***	LIR-9, ILT11, CD85f	Monocytes and neutrophils (28)	–	Macrophage activation, calcium flux regulation and production of pro-inflammatory cytokines (87)	–	RA (63) Allotransplantation (52)
***LILRA6***	ILT8, CD85b	Monocytes at mRNA level (88)	Cytokeratin-8 on necrotic glandular epithelial cells (89)	Macrophage activation, calcium flux regulation and production of pro-inflammatory cytokines (87)	High-grade serous ovarian cancer (20)	Atopic dermatitis (12) Allotransplantation (52)
***LILRB1***	LIR-1, ILT2, CD85j, MIR7	Monocytes, macrophages, DCs, T, B and NK cells (30), eosinophils (54), osteoclasts (90), placental mesenchyme (91), neutrophils, mast cell progenitors (12) and basophils (6, 41, 92, 93)	HLA-A, HLA-B, HLA-G (48), HLA-F (94) Homolog of HMCV UL18 protein (9) S100A8/9 (95) RIFIN (96) Dengue virus product (97), *S. aureus* and *E. coli* (98)	T cell inhibition, reduction of antigen recognition and release of anti-inflammatory cytokines (27, 99–105) B cell cycle arrest and inhibition (106, 107) DC inhibition (108, 109). NK cell cytotoxicity inhibition (110–113) Induction of tolerogenic DCs (27, 108, 114, 115) Macrophage differentiation and phagocytosis abrogation (116, 117)	CLL (112), glioma (118), AML (119), Burkitt’s lymphoma (107), gastric (120), lung (6, 121), renal (6), head and neck (6), esophagus (6), colon (6), liver (122, 123), breast (124), ovarian (125) and prostate (126) cancers	Bacterial infection (98), pulmonary tuberculosis (127), HIV (51, 95, 128–130), CMV (131, 132), Dengue virus (97), malaria (96, 133, 134), Zika (135), Epstein-Barr virus (136, 137) and chronic hepatitis B infection (138) MS (139), HT and GD (140), SLE (141–143), ankylosing spondylitis (12) and RA (17) Allotransplantation (52) and pregnancy (12, 92)
***LILRB2***	LIR-2, ILT4, CD85d, MIR10	Monocytes, macrophages, DCs (30), basophils (53), eosinophils (54), neutrophils (144), osteoclasts (90), placental vascular smooth muscle (91), platelets (145), neural cells, αβ oligomers, HSCs, endothelial cells, mast cell progenitors (12, 14, 41, 93), NK and T cells (41, 92)	HLA-A, HLA-B, HLA-G (48),HLA-F (94) ANGPTL 2 and 5 (146, 147) Cd1d, Cd1c, Nogo66, CSP, oligomeric β-amyloid (19, 148) UL18 (11) RTN4, MAG, OMgp (14, 41) RIFIN (92) SEMA4A (on activated CD4^+^ T cells) (149)	Induction of tolerogenic DCs and Tregs (27, 115, 150–158) Platelets inhibition (145) Activation and Th2 differentiation of CD4^+^ T cells (149) Supress monocyte-mediate pro-inflammatory response (97) Inhibition of monocyte differentiation into DCs (159) Impairment of neutrophil phagocytosis (144) Macrophage differentiation and phagocytosis abrogation (116, 117, 160, 161)	Hepatocellular carcinoma (82), PDA (82), AML (119), breast (162), lung (82, 121), colorectal (82, 163–165) and prostate (126) cancer	Pulmonary tuberculosis (166), Salmonellosis (56, 167), sepsis (144, 168), HIV (50, 128, 169, 170) and Zika (135) Alzheimer’s disease (160, 171, 172) and RA (63) Allotransplantation (52, 173) and pregnancy (12, 92)
***LILRB3***	LIR-3, ILT5, CD85a, HL9	Monocytes, macrophages, DCs (30), basophils (53), eosinophils (54), osteoclasts (90), neutrophils (23), B cells (174) and mast cell progenitors (12, 14, 92)	HLA I (α3 domain) (92) ANGPTL2 and 5 (146) Cytokeratin-8 on necrotic glandular epithelial cells (89) Complement components (148) *S. aureus (* 6) APOE4 (175)	Inhibition of basophil degranulation (53) Abrogation of IgA-mediated neutrophil phagocytosis, microbial destruction, and release of reactive oxygen species (176) Induction of immunosuppressive myeloid cells and inhibition of T cells (177)	AML (178) and colorectal cancer (179)	Bacterial infection (98, 176) RA (63) and TA (180) Allotransplantation (52, 181)
***LILRB4***	LIR-5, ILT3, CD85k, HM18	Monocytes, macrophages, DCs (30) osteoclasts (90), plasma cells, plasmoblasts (182), progenitor mast cells, microglia and endothelial cells (14, 23, 41, 93), T cells and neutrophils (6, 92, 183, 184)	APOE (185) ALCAM/CD166 (186) CNTFR (23, 41, 92) Fibronectin (14, 93, 187) Galectin-8 (188)	Induction of tolerogenic DCs and Tregs (27, 115, 150–158) Inhibition of cytokine production, activation and phagocytic activity of monocytes (14, 177, 189) Inhibition of T cell activity (185, 190)	CLL, AML (119, 185, 191, 192), multiple myeloma (193), hepatocellular carcinoma (122), melanoma (158), pancreatic (158), gastric (120), colorectal (194) (158) and lung (195, 196) cancer	Salmonellosis (56, 167) and COVID-19 (197, 198) MS (199, 200) and SLE (201) Allotransplantation (52, 173)
***LILRB5***	LIR-8, CD85c	Mast cells (intracellularly only) (202), monocytes, macrophages (14), T cells (6, 203), neutrophils, NK cells (23, 93), DCs and osteoclasts (41, 92)	HLA-B7 and HLA-B27 heavy chains (204) ANGPTL2 and 5 (146) Bacillus Calmette-Guérin (203)	–	–	–
***LILRP1***	ILT9, CD85l					
***LILRP2***	ILT10, CD85m					

AML, Acute Myeloid Leukaemia; AOSD, Adult-onset Still’s disease; CLE, Cutaneous Lupus Erythematous; CLL, Chronic Lymphocytic Leukaemia; CMV, Cytomegalovirus; COVID-19, coronavirus 19; GD, Graves’ Disease; HIV, Human immunodeficiency virus; HT, Hashimoto’s Thyroiditis; MS, Multiple Sclerosis; PDA, pancreatic ductal adenocarcinoma; RA, Rheumatoid Arthritis; SLE, Systemic Lupus Erythematosus; SS, Schrodinger’s Syndrome; TA, Takayasu’s Arteritis; -, Unknown.

Structural analysis of LILRB1-HLA I interaction has revealed that LILRB1 interacts with the highly conserved α3 β2m domains of HLA I, unlike T cell receptors (TCR) which bind to α1 and α2 domains, indicating they may bind simultaneously and demonstrating that LILRB1 may have a wider number of binding partners (35, 48). The interactions between LILRBs and HLA I may provide an inhibitory balancing force preventing immune activation to self and termination of immune responses.

The highest affinity LILR ligand is the non-classical HLA I molecule HLA-G, found in several forms including in disulphide-linked dimer or β2m-free isoforms. HLA-G interacts with LILRB1 and LILRB2 with different affinities (114, 205). LILRB1 lacks the reactivity to β2m-free HLA-G or HLA-B27, while LILRB2 interacts with the β2m-free form of HLA-B27 (48). Although LILRB2 exhibits overlapping HLA I recognition to LILRB1, it dominantly recognises the hydrophobic site of HLA-G D3 (48, 205). LILRB1 was shown to bind to HLA-G with 3 fold higher affinity compared to other HLA I molecules (150). In normal physiology HLA-G is expressed on foetal placental trophoblasts, enabling the invasion of the placental decidua during implantation and facilitating maternal tolerance to the semi-allogenic foetus (206). Enhanced expression of HLA-G contributes to the pathogenesis of viral infections and cancer by downregulating immune responses (207–213). Both HLA-G expression and dimerisation upregulate expression of LILRBs and inhibit T cell activity *in vitro (* 214, 215). Additionally, LILRB1 binds to various pathogens, including, *Staphylococcus aureus (S. aureus)* and *Escherichia coli (E. coli)*, opsonised dengue virus, cytomegalovirus (CMV), calcium-binding proteins S100A8 and A9 and repetitive interspersed family of polypeptides (RIFIN) (9, 19, 23, 94–98). LILRB2 binds to ANGPTLs (similar to LILRB3 and LILRB5), HLA I-like proteins, Nogo66, complement split products (CSP), oligomeric β-amyloid, UL18, RTN4, MAG, OMgp, RIFIN and SEMA4A in activated CD4^+^ T cells (11, 14, 19, 41, 92, 146–149). LILRB3 is the least studied LILRB and its natural ligands have not been fully elucidated. Although, regarded as an orphan receptor, recent findings suggest that LILRB3 interacts with ANGPTL2 and 5, complement components, and cytokeratin-associated proteins exposed on necrotic tumour cells and bacteria such as *S. aureus* (6, 23, 89, 92, 98, 146, 148). Hence, LILRB3 engagement by ligands expressed on necrotic cancer cells or pathogens may subvert immune responses. Recently, apolipoprotein (APOE) 4 was reported as a putative LILRB3 ligand, which is recognised by the D2/D4 regions LILRB3 (175). LILRB4 has been described to bind to APOE (185), ALCAM/CD166 (186), galectin-8 (188), CNTFR (23, 41, 92) and fibronectin (93, 187), while LILRB5 binds to ANGPTLs, HLA-B7 and HLA-B27 heavy chains and Bacillus Calmette-Guérin (146, 204).

The ligands for LILRAs are less characterised and may function as an autoregulatory mechanism for cell activation. They include HLA molecules for LILRA1, LILRA2 and LILRA3 (29, 48, 57, 64). Similar to LILRB5, LILRA1 binds to Bacillus Calmette-Guérin and also to *Mycobacterium bovis (* 49). Moreover, LILRA2 was shown to recognise IgG and IgM cleaved by proteases secreted by microorganisms such as *Mycoplasma hyorhinis*, *Legionella (L.) pneumophila*, *Streptococcus pneumonia* and *Candida albicans*. Interestingly, stimulation of primary monocytes via LILRA2 inhibited *L. pneumophila* growth (55). LILRA3 binds to Nogo66 (65), while LILRA4 binds to the bone marrow stromal cell antigen 2 (BST2) (79). However, there are no described ligands for LILRA5. Finally, LILRA6 is known to bind to cytokeratin 8 in necrotic glandular epithelial cells, similar to LILRB3 (89).

### 2.3 LILR signalling

LILRs signal via their associated ITAMs or ITIMs (**Figure 3**). As described above, LILRAs possess a transmembrane domain with a positively charged arginine and their short cytoplasmic domain has no kinase or docking motifs (30, 58, 81, 87). The arginine residue in LILRA2, LILRA4 and LILRA5 associate with a charged residue on FcεRIγ (58, 81, 87). Upon receptor crosslinking, Src kinases are activated and phosphorylate the ITAM tyrosine residues, which allows the phosphorylation of Src homology 2 domain (SH2) on Syk and ZAP70 tyrosine kinases (216) (**Figure 3**). ITAM-mediated signalling propagates the nuclear translocation of nuclear factor (NF)-κB and nuclear factor of activated T cells (NFAT), phosphoinositide (PI) 3-kinase (PI3K) activation, which activates membrane-bound serine/threonine-specific protein kinases (AKT and BTK), as well as interacting with Ras to activate the Ras/Raf pathway. As a result, ligation of LILRAs propagates the proliferation, maturation and survival of immune cells (217, 218) (**Figure 3**). The signalling mechanisms of LILRA1 and LILRA6 remain to be identified, although the structural similarity with the other LILRAs suggests that they also signal though FcεRIγ.

LILRBs impose their inhibitory signalling through ITIMs (30, 190). Upon receptor binding with the ligand, the Src-family protein Lyn becomes autophosphorylated, phosphorylating ITIM tyrosine residues. In turn, SH2 containing protein tyrosine phosphatases SHP-1 and SHP-2 are recruited to the phosphorylated sites. These phosphatases proceed to negatively regulate Syk and PI3K cell signalling (30). Consequently, downstream signalling pathways such as MAPK, JNK, Ras/ERK, NFAT and NF-κB are abrogated. This leads to attenuation of cytokine secretion and effector cell maturation, survival and function (218) (**Figure 3**). As an example, upon co-ligation of FcγRI with LILRB1/LILRB2, FcγR-mediated PTK-dependent signalling is abrogated (54, 116). Nevertheless, how multiple ligands and activating and inhibitory LILRs act in concert to modulate immune responses need further investigation.

### 2.4 LILR functions in leukocytes

**Figure 4:**
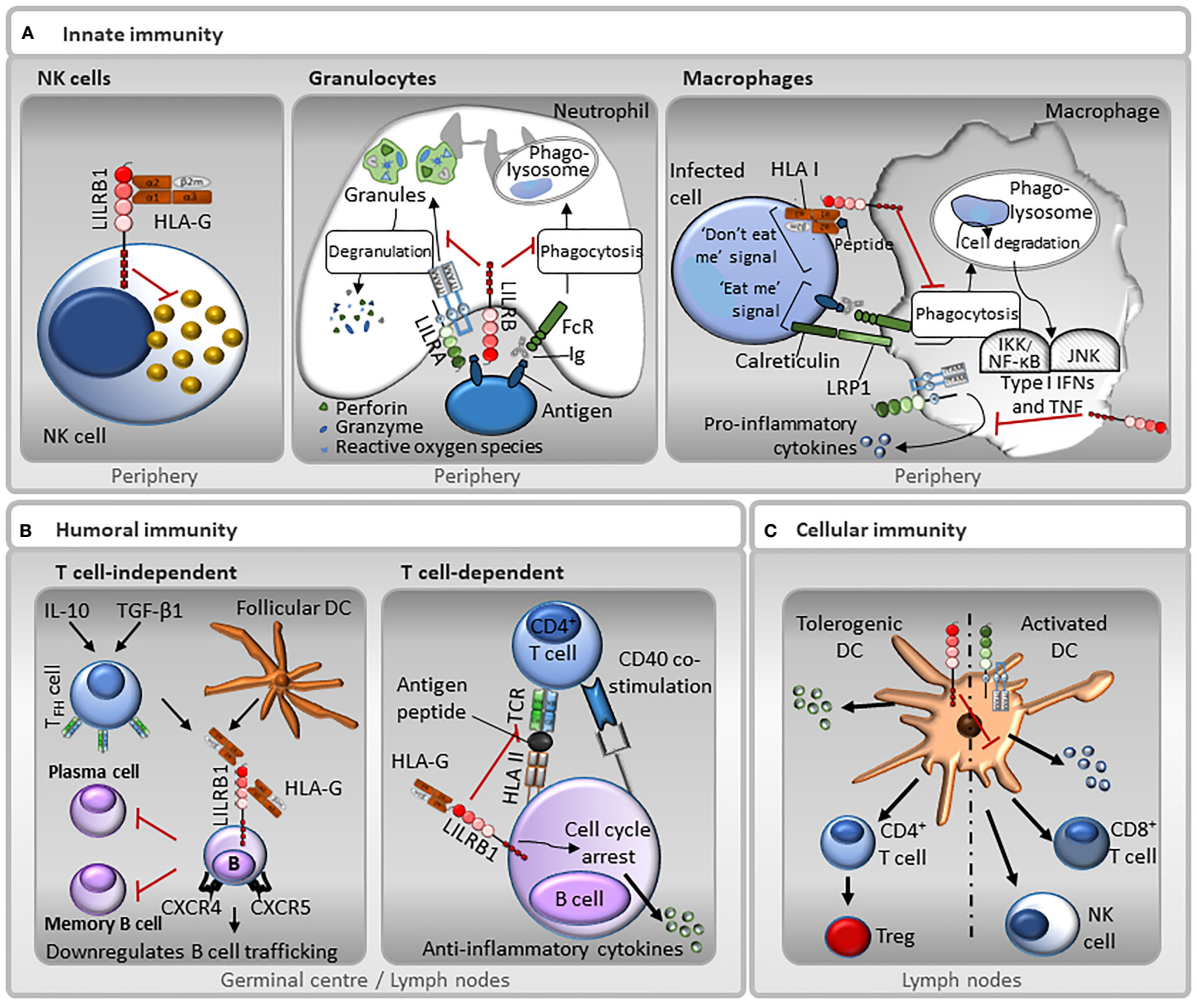
Representative functions of LILRs in innate and adaptive immunity. **(A)** *Innate immunity*: LILRB1 is expressed on NK cells, and therefore may be involved in missing self, whereby the receptor recognises HLA I molecules on target cells, and those that do not express HLA I are destroyed. In neutrophils, LILRAs, such as LILRA2 and LILRA5, stimulate degranulation and pro-inflammatory cytokines release, while LILRBs, such as LILRB3, have the opposite effect and block their phagocytic activity. Moreover, the paired-receptors LILRA6 and LILRB3 recognise bacterially-infected cells. Interaction between LILRBs, such as LILRB1, with HLA I abrogates FcγR-mediated phagocytic function of macrophages. In addition, LILRAs, such as LILRA3, LILRA5 and LILRA6 promote the secretion of pro-inflammatory cytokines. However, this secretion is suppressed by LILRBs. **(B)** *Humoral immunity*: LILRB1 inhibits B cell responses in a T cell-independent manner. TGF-β1 and IL-10 induce follicular dendritic cells (DC) and follicular helper T cells (TFH) to secrete HLA-G. HLA-G binds to LILRB1 on the surface of germinal centre B cells resulting in a down-regulation of chemokine receptors CXCR4 and CXCR5 and inhibiting B cell trafficking. This interaction also inhibits differentiation into antibody-secreting plasma cells and memory B cells. Moreover, LILRB1 regulates B cell responses in a T cell-dependent manner. B cells can present antigen to T cells. LILRB1-HLA-G interaction can prevent antigen presentation and inhibits B cell proliferation by causing cell cycle arrest in the G0/G1 phase by disrupting the mTOR pathway mediated by SHP-2. **(C)** *Cellular immunity*: Ligation of LILRBs during DC development renders DCs tolerogenic by increasing the threshold of activation. Normal DCs have low levels of LILRBs. In contrast, tolerogenic DCs that express increased levels of LILRBs, promote anti-inflammatory cytokines release, CD4^+^ helper T cells activation and their conversion into Tregs. Conversely, LILRAs activate DCs towards a cytotoxic phenotype, inducing the secretion of pro-inflammatory cytokines that induce NK and CD8^+^ T cells activation.

LILR functions have been primarily studied in terms of regulatory mechanisms exerted by LILRBs and, as such, little is known about the activating roles of LILRAs. Dividing the functions of LILRs as either activating or inhibitory, based on the presence of ITAMs or ITIMs may be too simplistic (219, 220). There have been suggestions that under certain conditions ITIM-bearing receptors can enhance leukocyte functions and ITAM-bearing receptors may inhibit the immune system (81, 217, 219–223). Based on the broad expression of LILRs across an array of immune cells and non-immune cells, their roles in controlling both innate and adaptive immunity are divided into leukocyte subsets herein, and the functional role of LILRs is discussed with regard to immune activation and tolerance (**Table 1**, **Figure 4**).

#### 2.4.1 Granulocytes

LILRAs are abundantly expressed on monocytes with some expression on granulocytes. LILRA crosslinking leads to cell activation resulting in calcium influx, selective cytokine release and degranulation (28, 53, 54, 58, 87). LILRA2, the most studied LILRA to date, as well as LILRA5, are implicated in stimulating degranulation and release of pro-inflammatory cytokines, such as IL-1β, IL-12 and tumour necrosis factor (TNF)-α and other factors involved in the early phases of eosinophils- and basophils-mediated inflammatory responses (54, 224). Microbially-cleaved Ig products activate primary human neutrophils via LILRA2 (55).

LILRB2, LILRB3 and LILRB5 are involved in regulating neutrophil activation and function (19, 23). LILRB2 is expressed on the surface of neutrophils as well as within the granules, inhibits their phagocytic capacity and leads to exocytosis of LILRB2 to the cell surface (144). This phenomenon of increased expression of inhibitory receptors upon activation may provide an inhibitory feedback loop (144). LILRB3 was recently found to be highly expressed on resting neutrophils and secreted upon their activation. Prolonged ligation of LILRB3 abrogated IgA-mediated neutrophil effector functions such as phagocytosis, microbial destruction and release of reactive oxygen species, suggesting that LILRB3 could be a novel checkpoint inhibitor on neutrophils (176). Similarly, co-ligation of PIR-B with FcεRI is able to abrogate IgE-mediated mast cell activation and serotonin secretion (42). A murine homologue of LILRB4, gp49B, is also expressed in mouse neutrophils and plays a regulatory role in lipopolysaccharide (LPS)-induced adhesion and microangiopathy (183, 184).

#### 2.4.2 Antigen-presenting cells

The expression of LILRs varies on macrophages and DCs at different maturation phases. DCs and macrophages detect surface microbial molecules through their pattern-recognition receptors, such as Toll-like receptors (TLR). However, APCs are also able to adopt a tolerogenic phenotype and orchestrate immune tolerance (150–153). Although LILRAs induce immune effector function, they can be inhibitory when ligated concurrently with an unrelated activating receptor on APCs (56, 217). Upon ligation of LILRA2 on monocytes, TLR-mediated antimicrobial activity was reduced by increased production of IL-10 (61). Furthermore, activation of LILRA2 on monocytes impaired their GM-CSF-mediated differentiation into immature DCs and supressed antigen (Ag) presentation and adaptive T cell response (59). In addition, LILRs can mediate cytokine secretion and affect the expression of co-stimulatory receptors on professional APCs. Accordingly, ligation of LILRA3 on monocytes and B cells increases the secretion of pro-inflammatory cytokines and indirectly induces proliferation of NK cells and CD8^+^ T cells (66). Similarly, LILRA2 ligation on monocytes is able to regulate TLR4 (56). Interestingly, both LILRA2 and LILRA3 inhibit LPS-mediated secretion of TNF-α by monocytes (63, 210). In addition, while treatment of monocytes with IL-10 and interferon (IFN)-γ increases secretion of soluble LILRA3, TNF-α reduces its expression (25, 225). Although less studied, LILRA4 inhibits the secretion of inflammatory cytokines by plasmacytoid DCs (pDC) (80). Furthermore, crosslinking of LILRA5 and LILRA6 on monocytes induces tyrosine kinase phosphorylation, which in turn mediates calcium flux and secretion of pro‐inflammatory cytokines (IL‐1β, TNF‐α, IL‐6), suggesting a potential role in inflammation. However, their underlying functions alongside LILRA1 remain unknown (87).

LILRBs can detect soluble factors including CSPs in response to microbial infections through classical lectin or alternative pathways of complement activation. Moreover, interaction of LILRB2 and C4d can suppress monocyte-mediated pro-inflammatory responses (97) and promote endocytosis of C4d (148). Tolerogenic APCs are unable to activate T cells, and they alternatively induce Ag-specific regulatory T (Treg) cells (153). Ligation of LILRB1 on monocytes during differentiation into DCs renders them tolerogenic (tDC), which in turn become resistant to LPS stimulation and unable to activate autologous T cells (27, 108, 114, 115). It also leads to increased expression of the NF-κB inhibitor ABIN1, key in maintaining functional DCs (226). Moreover, LILRB1 signalling inhibits DC activation mediated by OSCAR, which activates DCs via the FcRγ chain (108, 109). Banchereau et al. (227) showed that human Langerhans cells which do not express LILRBs were able to efficiently prime cytotoxic CD8^+^ T cells, whereas LILRB1- and LILRB2-expressing dermal CD14^+^ DCs were less efficient at priming cytotoxic T cells. Blockade of LILRB1/LILRB2 on dermal DCs enhanced T cell cytotoxicity (227). Similarly, tDCs exhibit high expression of LILRB2 and LILRB4, playing an essential role in tDC activity (27, 115, 150–153). Co-culture of T cells with an APC line transfected to express LILRB2 and LILRB4 extracellular domains demonstrated that only LILRB2 relies on its intracellular signalling to induce Tregs, whereas the extracellular Ig-like domains of LILRB4 and soluble LILRB4 were able to induce Tregs irrespective of their signalling potential (154, 155). Moreover, LILRB4 silencing in DCs promotes the release of pro-inflammatory cytokines and consequently the proliferation and migration of T cells (156). LILRB4 ligation on APCs leads to the upregulation of the co-stimulatory molecule CD86 (167), unlike LILRB2 which leads to its downregulation, indicating that LILRB2 and LILRB4 limit T cell responses via distinct mechanisms (150, 168, 169). In addition, LILRB2 and LILRB4 promote the differentiation of suppressor T cells (152, 155–158), and LILRB4 interacts with receptors on T cells and antagonises CD8^+^ T cells to promote the development of Tregs (154, 228). This suggests LILRB4 functions as an inducer of immune tolerance, while LILRB2 affects APC function and secondary co-stimulation via distinct mechanisms (150, 168, 169). Similarly, in PIR-B^-/-^ mice humoral T helper (Th) 2 responses are enhanced in response to T-dependent Ags due to impaired DC maturation compared to wildtype mice (229).

LILRB2-HLA-G interaction inhibits the differentiation of monocytes to DCs and maturation via IL-6 and STAT3 signalling (159). Moreover, LILRB4^-/-^ DCs stimulated via LPS-mediated TLR signalling exhibit increased pro-inflammatory cytokine/chemokine synthesis and secretion (156). Under physiological conditions, LILRB1 and LILRB4 are downregulated upon DC activation. This loss of inhibitory receptors may be essential for the maturation of DCs (230). Stimulation of DCs maturing *in vitro* with immunosuppressive agents, such as niflumic acid, IL-10, IFN-α and IFN-β, leads to the development of tDCs with increased LILRB2 and LILRB4 expression (27, 128, 151, 199, 231). Aspirin and 1,25-dihydroxyvitamin D_3_ (vitamin D) render DCs tolerogenic that are unable to stimulate T cells, only upon upregulation of LILRB4 (199, 232, 233).

LILRBs, therefore, act as myeloid checkpoint receptors to limit overt immune responses. LILRB1 ligation on tumour-associated macrophages (TAM) was shown to abrogate phagocytosis of HLA I^+^ tumours, which could be ablated with LILRB1 monoclonal antibodies (mAbs) (117). Similarly, combining LILRB2 and PD-1 blockade mediated *E. coli* phagocytic removal, which was associated with suppressed SHP1/2 phosphorylation, promoting pro-inflammatory macrophage activity (160). LILRB2 blocking reprograms macrophages to a more pro-inflammatory state and enhances the activation of T cells, increasing the efficiency of anti-PD-1 therapy (161). LILRB1 and LILRB2 co-ligation with FcγRI on monocytes mediates SHP-1 activation, abrogating downstream phosphorylation and intracellular calcium mobilisation (116). Ligation of LILRB3 on monocytes induces immunosuppressive myeloid cells, which inhibit T cell responses *in vitro*, and inhibit allograft rejection in humanised mice (177). Furthermore, LILRB4 activation results in recruitment of phosphatases that contribute to the dephosphorylation of FcγRI-activated tyrosine kinases and inhibition of FcγR-mediated phagocytosis (189).

Soluble isoforms of LILRBs (sLILRB), generated by alternative mRNA splicing, can also regulate immune responses. Jones et al. showed that sLILRB1 can compete with membrane-bound LILRB1 for binding to their natural ligands. This suggests that sLILRBs may act as decoy receptors in modulating immune effector functions (26). Similarly, recombinant sLILRB2 is able to restore the proliferation of T cells rendered inactive by tDCs (27). Notably, mature DCs treated with IL-10 abrogate shedding of sLILRB2, while increasing the expression of the surface-bound LILRB2 (27).

#### 2.4.3 NK cells

NK cells are cytotoxic against cells deficient for surface HLA I. Control of NK cell activation is regulated by activating and inhibitory receptors. Like KIRs, some LILRs can recognise HLA I molecules and influence NK cell effector functions. LILRB1 inhibits NK cell cytotoxicity as a result of HLA I interaction, which inhibits FcγRIIIA-dependent lysis of target cells (110). HLA-G-mediated LILRB1 ligation on NK cells inhibits activation, polarisation of lytic granules and IFN-γ production (111). LILRB1 blockade augments NK cell activation and proliferation and is associated with IL-2 production by CD4^+^ T cells (112). In addition, LILRB1 can regulate initial ligand recognition by abrogating the adhesion of NK cells to target cells (113).

#### 2.4.4 T cells

LILRB1 is the main LILR found on T cells, however, its expression is variable among CD8^+^ and CD4^+^ T cells, as not all T cells express LILRB1 (27, 99–104). LILRB1 abrogates TCR signalling by dephosphorylating the TCR-ζ chain of ITAM domains, which in turn suppress downstream signal transduction mediated by ZAP70 and linker for activation of T cells (105). LILRB1-mediated inhibition of T cells is characterised by a reduction in Ag recognition, CD3-mediated clonal expansion, proliferation, chemotaxis, resistance to TLR stimulation and a shift in the cytokine profile in favour of anti-inflammatory cytokines (27, 99–104). As CD8^+^ T cells mature, they acquire cytotoxic potential with an increase in perforin within the cell; LILRB1 expression increases in parallel, possibly to protect self (136). Crosslinking of surface LILRB1 or CTLA-4 on T cells leads to inhibition of Ag-specific CD4^+^ T cell proliferation, and IL-13, IFN-γ and IL-2 release, as well as an increase in TGF-β and IL-10 secretion (99). Apart from mediating inhibitory signals to T cells (111, 234), both surface-bound and soluble forms of LILRB1 and LILRB2 limit activating signals by antagonising the HLA I-CD8 interactions (228). Moreover, LILRB2 is expressed in CD4^+^ T cells and regulates Th2 differentiation (149). Also, LILRB4 has been found expressed on T cells, and it suppresses T cell activity mediated by APOE/the intracellular domain of LILRB4/SHP-2/NF-κB/urokinase receptor/arginase-1 (ARG1) axis (185, 190). Finally, there are contradictory reports on whether T cells express LILRB5 or not, which may be due to the nature of the reagents and assay conditions used by the investigators (203, 204). Additionally, LILRA2 has been found on T cells at low levels (56), and regulates T cells indirectly by modulating the behavior of other cells, such as APCs (59).

#### 2.4.5 B cells

Some LILRs are expressed by B cells but they primarily impact B cell responses by modifying APC and T cell responses (235). Transcripts of LILRA1, LILRA3 and LILRB3 are found in B cells, and LILRB4 is present in plasmablasts (11, 174, 182). However, only LILRB1 has a clear role in B cells. HLA-G binding to LILRB1 on B cells inhibits both T cell-dependent and -independent activation of naïve and memory B cells (106). Furthermore, LILRB1 interaction with HLA-G leads to B cell G0/G1 cell cycle arrest as a result of mTOR/AKT and PKC pathway modification (106). LILRB1-ligated B cells exhibit reduced Ig secretion and increased secretion of anti-inflammatory cytokines (106, 107).

### 2.5 The clinical relevance of LILRs

#### 2.5.1 Infection

Apart from the pivotal roles of LILRs in maintaining immune homeostasis, they can mediate pathogenesis during bacterial, viral and parasitic infections, as extensively reviewed elsewhere (49). Below is a summary of their key roles in infection.

##### 2.5.1.1 Bacterial infections

*Mycobacterium leprae* infection mediates strong Th1 cell-mediated immune responses, to give rise to the tuberculoid form of leprae. In contrast, lepromatous leprosy infection involves higher bacterial load, dominance in Th2 cytokine secretion and strong humoral immune responses (61). LILRB3, LILRB5 and especially the activating LILRA2, are overexpressed in skin biopsies from patients with lepromatous leprosy, which is associated with inhibition of TLR-mediated microbial killing, secretion of type 2 cytokines with an increase in IL-10:IL-12 ratio (61). Genetic profiling and immune labelling of skin lesions of these two forms has revealed a substantial regulation of LILRA2 on the disseminated lepromatous leprosy lesions over the limited tuberculoid form. Pre-treatment with LILRA2 antibodies reduces TLR-mediated antimicrobial activity (59, 61). In the case of *Mycobacterium tuberculosis*, its major niche to persist are macrophages and myeloid-derived suppressor cells (MDSCs). Blocking LILRB2 reprograms myeloid cells to be more pro-inflammatory and enhances the killing of intracellular *Mycobacterium tuberculosis*(166). Moreover, patients with active pulmonary tuberculosis have a higher frequency of LILRB1^+^ CD56^dim^ FcγRIIIA^+^ NK cells, which correlates with disease severity (127). Conversely, recent data implicates LILRA2 in pathogen sensing and activation of innate immunity against microbial pathogens via the recognition of cleaved IgM and IgG products by proteases from *S. pneumonia*, *Legionella pneumophila*, *Mycoplasma hyorhinis* and *Candida albicans*(55). Neutrophils and monocytes expressing LILRA2 are activated by these cleaved Igs, enhancing immune responses against these bacteria (55).

Infection with *Salmonella typhimurium* can modulate APCs, especially macrophages and DCs. Exposure of APCs to *Salmonella* mediates upregulation of LILRB2 and LILRB4 and downregulation of LILRA2. This tuning in the balance of the LILR family members suppresses innate immune responses by increasing the IL-10:IL-12 ratio (56, 167). Mouse fibroblast cells generated to express PIR-B, LILRB1 or LILRB3 are all able to recognise Gram positive *S. aureus*, while LILRB1 is also able to bind *E. coli*(98). LILRB3 was recently reported to inhibit neutrophil effector functions and microbial killing, whereby ligation of LILRB3 abrogated IgA-mediated phagocytic uptake, reactive oxygen species generation and microbial killing of *S. capitis*(176).

The key pathogenic element of sepsis is systemic inflammation. However, most patients suffer signs of severe immunosuppression and fail to address the primary bacterial infection. Immune dysregulation during sepsis is associated with increased LILRB2 expression on monocytes and organ dysfunction (168). LILRB2^+^ monocytes express lower levels of CD86 and have an increase in IL-10:IL-12 cytokine ratio (168). In addition, LILRB2 upregulation found on healthy donor-derived activated neutrophils is impaired in septic patients with a consequent inhibition of their phagocytic function, proposing LILRB2 as a therapeutic target to prevent neutrophil dysfunction and exacerbated inflammation (144). Additionally, the antibiotic amoxicillin binds to HLA I, increasing NK cells cytolysis due to the inhibition of LILRB1 binding (236).

PIR-A and PIR-B are able to recognise cell wall components of both Gram positive and negative bacteria (98). Wildtype mice exhibit greater mortality than PIR-B^-/-^ mice upon *S. aureus* infection. Stimulation of macrophages from PIR-B^-/-^ mice with *S. aureus* results in increased levels of TLR-induced inflammatory cytokines IL-6 and IL-1β, compared to wildtype macrophages (237). Moreover, PIR-B is upregulated on macrophages after LPS treatment and negatively regulates the secretion of pro-inflammatory cytokines during *E. coli* infection (238).

Bacterial infections result in overexpression of most of the LILRBs, modulating leukocytes to a more anti-inflammatory state and blocking their effector properties. In contrast, LILRAs have an opposite role, depending on the type of infection.

##### 2.5.1.2 Viral infections

Viruses interact with LILRs to suppress antiviral responses (9). The CMV gene product UL18 binds to LILRB1 on DCs rendering them resistant to maturation signals and unable to activate naïve T cells (115, 239), potentially so that CMV-infected cells can avoid elimination (9, 240). Analysis of memory T cells from CMV patients found high LILRB1 expression, with levels appearing to increase over time (241). Additionally, lung transplant recipients with elevated levels of LILRB1 on lymphocytes are at increased likelihood of CMV infection (131). However, investigations into the role of the UL18-LILRB1 interaction on T cells has yielded contradictory results. One study found that LILRB1 on cytotoxic T cells mediates lysis of virally-infected cells expressing UL18 independently of TCR, while cells infected with human CMV defective for UL18 were not lysed (132). In contrast, others have demonstrated that UL18 protects infected cells from LILRB1^+^ NK cell cytolysis. This protection was abrogated if cells were infected with CMV containing an UL18 mutant. In addition, UL18 mediated the activation of LILRB1^-^ NK cells, which can mask LILRB1^+^ NK cell inhibition (242). Moreover, LILRB1 is highly expressed on viral-specific CD8^+^ T cells in Epstein-Barr virus-infected individuals (136, 137). LILRB1 expression is elevated on viral-specific CD8^+^ T and NK cells and interacts with viral products to downregulate immunity (129, 136, 137). NK cell activity was impaired in patients with chronic hepatitis B. Circulating CD56^dim^ FcγRIIIA^+^ NK cells had increased LILRB1 in immunotolerant patients, which positively correlated with their serum viral load. Interestingly, LILRB1^+^ CD56^dim^ NK cells were reduced with antiviral therapy, and LILRB1 blockade increased their cytotoxicity (138).

The interaction between LILRs and HIV infection is emerging as an important determinant of HIV progression (50). Upon HIV infection, DC dysfunction correlates with the upregulation of LILRB1 and LILRB2 (128) and downregulation of LILRA1 and LILRA2 (50, 51). In these patients, LILRB1 is upregulated on CD8^+^ T and NK cells, while LILRB2 expression is increased on myelomonocytic cells due to the increase in IL-10. These monocytes are defective in Ag presentation, which in turn abrogates the antiviral T cell responses and CD4^+^ T cell proliferation (128). These results indicate that the presence of high IL-10 levels in the sera of HIV^+^ patients impede Ag presentation of APCs by increasing LILRB2 expression. More recently, LILRB2 affinity for HLA I molecules was shown to positively correlate to the viral load in the majority of untreated HIV-1 patients. DCs in this cohort of patients were shown to have impaired Ag presentation ability as a result of LILRB2 crosslinking by HLA I molecules (170). In contrast, LILRBs can also enhance APC activity to stimulate T cells from HIV-1 elite controllers (51). These DCs express elevated levels of LILRB1 and LILRB3, blockade of which diminishes the enhanced Ag presenting properties (51). This enhanced T cell stimulating ability is contrary to other *in vitro* studies, which demonstrate that LILRB1 reduces the capability of DCs to stimulate T cells (115, 239). The interactions between LILRs on immune cells and HLA I expressed on HIV-infected cells is important to the response against infection (50). Specific HIV escape mutations when loaded as epitopes on HLA I diminish recognition by TCRs and enhance binding to LILRB2, resulting in the development of tolerogenic myelomonocytic cells (50, 169). Moreover, HLA-G is elevated in sera, and on monocytes and T cells of HIV-infected individuals (209, 243, 244). LILRB1 has been found overexpressed on NK cells after HIV-1 infection and these LILRB1^+^ NK cells control virus replication in DCs (130). However, the same laboratory has demonstrated that the inflammatory protein S100A9 expressed on HIV-infected DCs interacts with LILRB1 on NK cells and reduces DC cytotoxicity despite increased TNF-α secretion (95). These discrepancies potentially relate to different virality and stage of the disease (129).

pDCs are the only cell type known to express LILRA4 and are important in innate responses to viruses and tumours, producing significant quantities of IFNs following TLR7 and TLR9 ligation (78, 79, 81, 245). Indeed, LILRA4 is used as a marker of pDC subpopulations in coronavirus-19 (COVID-19)-infected patients (246), an APC subset that is reduced in severe cases (86). The only known ligand for LILRA4 is BST2, which prevents prolonged IFN production and assures TLR response by pDCs. BST2 expression is stimulated on a variety of cells by IFN and TLR7/9 ligands and is elevated during HIV infection (79, 247). The ability of IFN to induce BST2, which in turn interacts with LILRA4 to downregulate the IFN-producing pDCs, may serve as a negative feedback loop limiting IFN production (79).

Dengue virus is able to facilitate infection of myeloid cells by using antibody opsonisation to bind to activating FcγRs (97). Crosslinking of activating FcγRs leads to the induction of type-1 IFNs though Syk signalling, responses potentially deleterious to the internalised virus. To avoid this, viral proteins co-ligate LILRB1 on myeloid cells, which recruit phosphatases to inhibit Syk, preventing productive signalling. However, the ligand of LILRB1 on dengue virus remains unknown (97). In COVID-19 patients, LILRB4 expression is linked to disease severity and associates with a strong expansion of MDSCs and poor T cell responses, increasing immunosuppression (197, 198). Similarly, polymorphisms in LILRB1 and HLA-G are linked to higher risk of Zika virus transmission from mother to foetus, while certain polymorphisms in LILRB2 have a protective function (135).

Interestingly, the D3-4 region of PIR-B has been recently described to bind reovirus, allowing infection and producing serotype-dependent neuropathogenesis in infected mice (248).

##### 2.5.1.3 Parasitic infections

Infection with the parasite *Plasmodium falciparum*, which develops into malaria, is associated with inflammatory cytokine production. LILRB1 has been shown to be upregulated on apoptotic B cells in the peripheral blood of patients with severe malaria compared to healthy controls. These early apoptotic LILRB1^+^ CD19^+^ B cells contribute to the inflammatory cytokine storm and impairment of immune memory (249). RIFINs, which are the causative targets of the malarial parasite, act as ligands for inhibitory receptors. A recent study proposed that LILRB1-binding RIFINs mimic the binding interface of the natural ligands of LILRB1 at the immunological synapse of NK cells, which suppresses NK cell cytotoxicity (96). LILRB2 also binds to RIFIN expressed on *Plasmodium falciparum*-infected erythrocytes, proposing it to produce a similar immune evasion to LILRB1 (133, 134). Additionally, infection with *Toxoplasma gondii* during pregnancy provokes a downregulation of LILRB4, switching macrophages and decidual MDSCs to a more pro-inflammatory state, contributing to adverse outcomes during pregnancy (250). In contrast, there is an upregulation of LILRB2 in non-classical monocytes of infants born to placental malaria mothers, enhancing susceptibility to the disease (251).

#### 2.5.2 Autoimmunity and neurodegenerative disorders

The immunomodulatory capacity of LILRs has been associated with autoimmune diseases and neurodegenerative disorders (**Table 1**). However, the functions of LILRs in these settings have not been fully elucidated.

##### 2.5.2.1 Thyroid disease

Hashimoto’s thyroiditis (HT) and Graves’ disease patients express elevated levels of LILRB1 on peripheral CD4^+^, CD8^+^ and NK cells as well as thyroid tissue (HT patients). However, stimulation of these cells *in vitro* in the presence of a LILRB1 mAb has revealed that the receptor has an attenuated and defective ability to inhibit T cell proliferation. This reduced activity was mediated by IL-10 and contributed to poor control of inflammation in autoimmune disease (140).,

##### 2.5.2.2 Multiple sclerosis

Two studies looking at a western European population found an association between the deletion of *LILRA3* and an increased risk of multiple sclerosis (MS), whereas a study of a Polish population found that *LILRA3* deletion was associated with later onset of MS (68, 74–76). In patients with MS, abundant expression of HLA-G and LILRB1 in areas of activated microglia, central nervous system (CNS) phagocytic cells, and periplaque tissues indicates that LILRB1-HLA-G interaction can regulate immune homeostasis of the CNS (139). Furthermore, LILRB4 is downregulated on monocytes during MS relapse (200). MS patients treated with IFN-β and vitamin D_3_ exhibit DC tolerance, in a LILRB4-dependent manner (199, 200). In addition, in the experimental autoimmune encephalopathy (EAE) mouse model of MS, sLILRB4 binds to immune cells and reduces the secretion of pro-inflammatory cytokines, delaying the evolution of the disease (252). Interestingly, it has been reported that glatiramer acetate (GA), a therapeutic molecule for relapsing-remitting MS, interacts with PIR-B on MDSCs and reduces pro-inflammatory responses. In addition, soluble GA competitively interacts with LILRB2 and LILRB3, modulating the alternative activation of monocytes and macrophages (253).

##### 2.5.2.3 Alzheimer’s disease

LILRB2 and PIR-B bind to oligomeric β-amyloid forms, which are involved in memory deficits and loss of synaptic plasticity. Interestingly, PIR-B-deficient mice do not have signs of damage caused by β-amyloid peptide or synaptic loss, implying its role in β-amyloid-induced Alzheimer’s disease (171, 172). Hence, many efforts have been made to improve synapsis elimination by disrupting LILRB2-β-amyloid interactions, for instance, with structure-guided small molecule inhibitors that physically impede the binding (254).

##### 2.5.2.4 Systemic lupus erythematosus and cutaneous lupus erythematous

Examination of peripheral blood mononuclear cells (PBMC) from systemic lupus erythematosus (SLE) patients has revealed reduced expression of LILRB1 on CD4^+^ and CD8^+^ T cells, B cells and DCs, with LILRB1 on these cells demonstrating a diminished inhibitory function compared to healthy donors (141, 142). Moreover, LILRs possess high levels of polymorphisms that have been implicated with different autoimmune disorders, including SLE. A splice-site single nucleotide polymorphism (SNP) in *LILRA2* gives rise to novel isoforms expressed on monocytes and is associated with higher susceptibility to SLE and microscopic polyangiitis (62). Furthermore, high expression and functionality of *LILRA3* are associated with higher susceptibility to SLE and an increased disease activity and severity when induced in CD14^+^ monocytes (69, 71). Specific SNPs within *LILRB4* observed in SLE patients are associated with its decreased surface expression on DCs, further correlating with increased serum type I IFNs and TNF-α (201). These results suggest that LILRBs have a potential role in the pathogenesis of SLE. LILRA4, BST2 and type I IFNs are orchestrated in a loop that regulates pDCs activation (24, 79). The release of autoantigens from dying keratinocytes induces neutrophil extracellular traps (NET) that promote the activation of LILRA4-expressing pDCs. This persistent activation drives the release of type I IFNs, provoking cutaneous lupus erythematous (CLE). Type I IFNs are increased in CLE patients (83) and CLE is known as a type I IFN disease (84, 85). Hence, LILRA4 has been studied as a specific target for some autoimmune disorders (255).

##### 2.5.2.5 Sjogren’s syndrome and Takayasu’s arteritis

Genotyping studies suggest that patients homozygous for *LILRA3* deficiency exhibit higher susceptibility to Sjogren’s syndrome (SS) (68–70). LILRA3 shares close homology with LILRA2, LILRB1 and LILRB2, so it may bind to their ligands either agonistically or antagonistically, possibly accounting for the contrary associations with LILRA3 in inflammatory diseases (25, 200). A risk allele (rs103294) in *LILRA3* is involved in the deletion of the gene, and the epistasis of *LILRA3* and HLA-B*52 might play an important role in Takayasu’s arteritis (TA), possibly by over activating NK cells (256). However, deeper analyses are needed to confirm an actual correlation. Additionally, genome-wide association studies of TA patients have identified a SNP which is associated with reduced *LILRB3* expression as a susceptibility allele (180).

##### 2.5.2.6 Adult-onset Still’s disease

Neutrophil activation with high degree of NET formation is associated with the pathogenesis of adult-onset Still’s disease (AOSD). In a recent study, *LILRA3* was reported to act as a novel genetic risk factor for AOSD, with elevated plasma LILRA3 levels in AOSD patients. NET formation was enhanced in neutrophils from AOSD patients upon LILRA3 stimulation (77).

##### 2.5.2.7 Rheumatoid arthritis

Aberrant expression of LILRs has been associated with several arthritis syndromes. LILRA2, LILRA3, LILRA5, LILRB2, and LILRB3 are found at elevated levels in the sera and synovial fluid of RA patients, correlating with disease severity (25, 63, 72, 73, 87, 257). Significantly lower numbers of LILRA2^+^, LILRB2^+^ and LILRB3^+^ inflammatory cells were detected in RA patients who responded to anti-rheumatic therapy compared to healthy controls, as a result of the partial blocking of LILRA2-mediated secretion of TNF-α (63, 87, 257). Additionally, LILRA3 promotes pro-inflammatory responses in fibroblast-like synoviocytes, promoting their activation, migration and invasion *in vitro* (73). Anti-rheumatic drugs downregulate synovial expression of LILRB2, LILRB3 and LILRA2 in responding patients. However, this is not replicated *in vitro*, suggesting that the drugs do not act directly to impact LILR expression (257). While LILRA2 and LILRA5 are expressed highly in patients treated with methotrexate, LILRB2 is elevated in patients treated with prednisone (anti-inflammatory) (63). The potential relevance of these receptors in rheumatic inflammation is underlined by the ability of LILRB1, LILRB2 and LILRA2 to engage with HLA-B27, a haplotype associated with several inflammatory diseases. LILRB2 has been implicated in the pathogenesis of spondylarthritis, since LILRB2 can recognise several HLA-B27 isoforms and regulate innate and adaptive inflammatory responses (48). Moreover, LILRB1 binds to sHLA-G in RA patients protecting them against inflammation. However, this binding is not seen in advanced RA patients with long-term chronic inflammation, which impedes the immunosuppression and reduction of inflammation mediated by LILRB1 (258). Due to the high polymorphic nature of *LILRs*, different alleles can confer susceptibility to RA. A haplotype of *LILRB1* that leads to reduced surface expression of the receptor is associated with high susceptibility to RA in HLA-DRB1 shared epitope-negative patients, possibly because of insufficient inhibitory signalling in their leukocytes (17).

#### 2.5.3 Allotransplantation and pregnancy

APCs derived both from the recipient and donor are able to present Ags to T cells, and play a key role in transplant immunity (259). Consequently, alloreactive T cells are stimulated, which result in allogeneic graft rejection. Elevated levels of circulating T suppressor cells, tolerogenic APCs and HLA-G augment immunosuppression and are associated with a more favourable allotransplant acceptance (153, 260, 261). As receptors for HLA-G, LILRBs can be considered as therapeutic targets for medicating transplantation tolerance. In an organ transplantation setting, LILRB-mediated inhibition of T cells induced immune tolerance to allow allograft acceptance (249). LILRB1, LILRB2 and LILRB4 play fundamental roles in the immunosuppression cascade (**Figure 4**). Rejection-free heart, liver and kidney transplant recipients all possess alloantigen-specific CD8^+^ T suppressor cells (153, 260, 261). These T cells are able to induce LILRB2 and LILRB4 expression on donor DCs and monocytes and abrogate the expression of CD80/CD86 co-stimulatory molecules and alloreactive CD4^+^ Th cell proliferation (153, 260, 261). T suppressor cell-mediated tolerance extends to non-professional APCs including donor endothelial cells to confer tolerance of APCs (262, 263). In addition, *LILRB1* was found highly expressed in circulating non-classical and intermediate monocytes of kidney transplant recipients. Interestingly, myeloid cells from kidney biopsies showed an upregulation of *LILRB1*, *LILRB2* and *LILRB3* after antibody-mediated rejection (ABMR), whereas circulating non-classical monocytes specifically had higher levels of *LILRB3* and *LILRB4* after ABMR (52).

Transplanted human pancreatic islet cells are tolerated by PBMC-engrafted NOD/SCID mice when treated with sLILRB4. This graft acceptance is associated with expansion of CD8^+^ T suppressor cells and diminished Th reactivity against graft HLA alloantigens (158, 264). The immunosuppression induced by the drug rapamycin is associated with increased LILRB2 and LILRB4 expression on DCs and a related increase in Tregs, T suppressor cells and serum HLA-G (173). Moreover, interaction of LILRB1 and LILRB2 with soluble and membrane-bound HLA-G from transplant patient sera augments Tregs and MDSCs and reduces T cell proliferation, enhancing the survival of skin allograft (215, 265–273). Similar findings were demonstrated in animal studies, where PIR-B was shown to enhance allotransplant acceptance. UVB-irradiated DCs that were unable to stimulate CD4^+^ T cells induced tolerance in heart transplant recipient rats, characterised by T suppressor cells and upregulation of PIR-B on APCs. Re-transplantation of PIR-B^+^ APC heart allografts to a second recipient failed to elicit rejection, indicating that these PIR-B^+^ APCs are responsible for tolerance (274). In addition, LILRB2^+^ DCs in LILRB2 transgenic mice induced tolerance against skin allografts when treated with HLA-G microbeads, via STAT3 and IDO activation and T cell suppression (273, 275, 276). HLA-G treatment of LILRB1 transgenic mice that previously received allogenic skin grafts resulted in expansion of MDSCs, which was associated with prolonged allograft survival (271).

Graft-versus Host Disease (GVHD) is the foremost impediment of allogeneic hematopoietic stem cell transplantation (HSCT), which in turn is associated with rejection of the allograft. PIR-B-deficient mice that received allogeneic T cells exhibited aggravated GVHD compared to wildtype mice as a result of the stimulation of PIR-B-deficient DCs (277). Similarly, acute GVHD was abrogated in mice that received PIR-B-transfected DCs, which were deficient in CD80/CD86 co-stimulatory molecules (278). A clinical study reported that 5.4% of patients that received HSCT, but not solid organ, had LILRB3-reactive antibodies directed against LILRB3^+^ DCs. These patients also expressed LILRB3 on leukemic cells, proposing LILRB3 as a GVH and graft-versus-leukaemia target (181). Moreover, our group demonstrated that mAb-mediated ligation of LILRB3 in humanised mice induces tolerance, allowing the engraftment of allogenic lymphoma cells (177). Collectively, these studies demonstrate that LILRBs are key regulators of immune tolerance and allograft acceptance and present an exciting therapeutic opportunity. Contrary to LILRBs, few studies have analysed the role of LILRAs in allotransplantation. A recent study showed that *LILRA1* was highly expressed in circulating FcγRIIIA^+^ CD14^-^ non-classical monocytes after kidney transplantation. Additionally, *LILRA5* and *LILRA6* were found overexpressed in circulating non-classical monocytes after ABMR and *LILRA5* was highly expressed in myeloid cells from kidney tissues (52).

Pregnancy can be considered a type of allotransplant. Although the mechanisms that prevent foetal rejection by the maternal immune system remain incompletely known, LILRs have been implicated. HLA-G is expressed in trophoblasts during pregnancy, hence its interaction with LILRBs is considered essential (92). During pregnancy LILRB1 ligation inhibits the cytotoxicity of NK cells, while LILRB2 promotes M2 macrophage polarisation and MDSC suppressive activity (12, 92). Moreover, LILRB2 promotes DC tolerance and MDSC activation by binding to sHLA-G, and both LILRB1 and LILRB2 regulate B and T cell functions to maintain pregnancy. Considering all of this and their relevant role in placental vascular remodelling and foetal development, LILRBs are being considered as biomarkers of recurrent implantation failure (273, 279).

#### 2.5.4 Cancer

In addition to expression on immune cells and their dysregulation within the tumour microenvironment (TME), LILRs may also be present on cancer cells, to support tumourigenesis and suppress anti-tumour immunity. Hence, LILRs may be exploited as potential targets in cancer immunotherapy. It is now appreciated that LILRs may play central roles in a number of hallmarks of cancer: immune-evasion, inflammation, tumour cell proliferation and metastasis (6, 41).

##### 2.5.4.1 LILRAs and cancer

The implication of LILRAs in cancer has not been fully addressed. In oestrogen receptor-positive breast cancer patients, *LILRA2* gene expression correlated with tumour shrinkage (60), and in pancreatic ductal adenocarcinoma (PDA) higher transcript levels were associated with relapse-free survival (82). In the case of LILRA3, its ligation on monocytes was proposed to stimulate CD8^+^ T cells and NK cells *in vitro*, suggesting LILRA3 may be immunostimulatory (66). In addition, genetic deletion of *LILRA3* leads to predisposition to non-Hodgkin’s lymphoma (66), while its presence is more common in prostate cancer patients of Chinese Han origin than in healthy controls (67). Notably, LILRA4 ligation can inhibit IFN-α and TNF-α production from pDCs. pDC infiltration in human tumours has been associated with poor prognosis, linked to impaired ability to produce the tumouricidal IFN-α (80, 280). More recently BST2 has been identified as a ligand for LILRA4, which is also expressed on several human cancers and downregulates IFN-α production, implying a mechanism through which tumours interact with LILRA4 to suppress immunity (79, 245). Additionally, a study looking at PDA patients found that higher *LILRA4* expression is associated with better overall survival (OS) (82). Finally, a genome-wide association study showed that duplications at *LILRA6* were associated with high-grade serous ovarian cancer susceptibility (20).

##### 2.5.4.2 LILRBs and cancer

**Figure 5:**
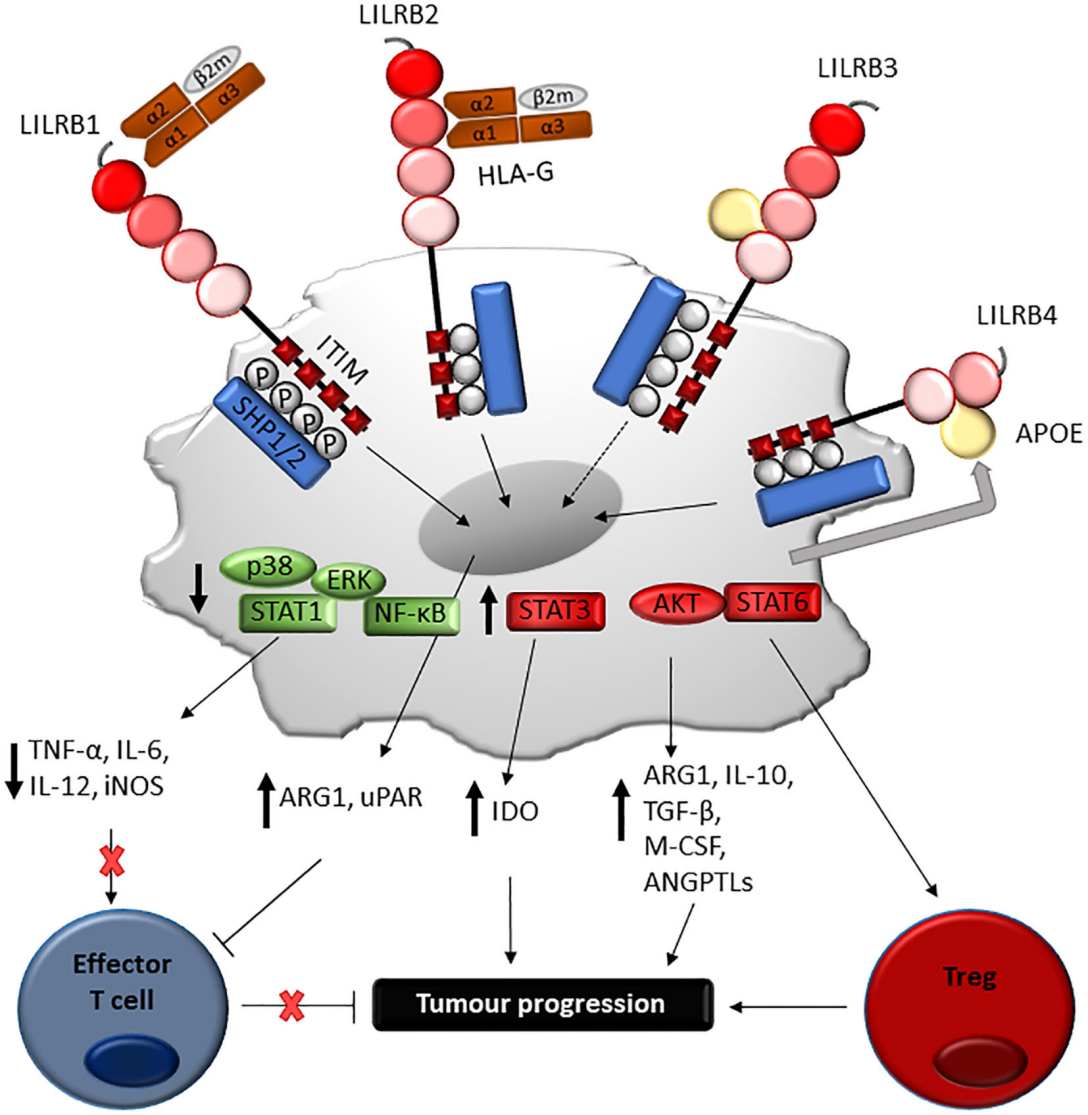
Proposed mechanism of LILRB-mediated immune-evasion and tumour progression via myeloid cells. Engagement of LILRB1 and LILRB2 with HLA-G on myeloid-derived suppressor cells (MDSC) activates STAT6 and STAT3-mediated cascades, which in turn induces ARG1 and IDO production responsible for T cell suppression. LILRB4 ligation by APOE on monocytic AML cells mediates SHP-2 inhibitory signalling, which in turn positively regulate the NF-κB pathway. This leads to ARG1 production and urokinase-type plasminogen activator receptor (uPAR), responsible for T cell suppression and support of leukaemia migration. Although further studies are needed, LILRB3 ligation can mediate similar processes, such as induction of amphiregulin.

In contrast to LILRAs, there is compelling evidence that LILRBs are implicated in tumourigenesis, as well as tumour immune-evasion and progression. Examination of human cancer cell lines and tumour specimens has highlighted three main mechanisms. Firstly, aberrant LILRB expression occurs in several human cancers but not healthy adjacent tissues, with the expression of LILRBs and HLA-G found to correlate with poorly differentiated, more advanced or aggressive cancers in most cases (80, 120, 121, 124, 206–208, 210, 211, 213, 281–283). Secondly, IL-10 contributes to the LILRB : HLA-G axis of immunosuppression, as it upregulates LILRB and HLA-G (27, 98, 114, 115, 128, 143, 151, 282). Thirdly, recent advances have implicated LILRB signalling and expression directly with tumour progression and worse therapeutic response (119, 284). Directly or indirectly, many important LILRB functions involve modulation of myeloid cells (**Table 1**, **Figure 5**). Since the role of LILRBs in cancer have recently been reviewed elsewhere (6, 41, 93, 249), a brief summary is outlined here.

LILRB1 is found on a variety of cancers including breast and prostate cancers, hepatocellular carcinoma, B cell lymphoma, acute myeloid leukaemia (AML), acute lymphoblastic leukaemia (ALL) and gastric cancer cell lines (110, 120, 124, 126). Moreover, a pan-cancer genomics analysis showed that *LILRB1* was highly mutated in various cancers (119). In addition to expression on tumour cells, higher levels of LILRB1 have been demonstrated on the peripheral blood of non-small cell lung cancer (NSCLC), renal, head and neck, oesophagus and colon cancer patients than healthy individuals (6). Histological analysis of breast cancer biopsies revealed LILRB1 expression on CD68^+^ macrophages and CD8^+^ T cells (124). There is strong evidence that LILRB1 mediates cancer immune-evasion. Additionally, LILRB1 has been recently defined as a prognosis marker in ovarian cancer; high expression correlating with immunosuppression and its levels on immune cells associating with the clinical subtype and stage, resistance to platinum treatment and PD-1/PD-L1 mAb therapy (125). LILRB1 and LILRB4 are also expressed on human primary gastric cancer specimens compared to healthy tissue, with high expression correlating with advanced disease (120). Expression of LILRB1 on gastric cancer cell lines induces resistance to NK cell cytotoxicity (120, 283). Interestingly, LILRB1 gene and protein levels correlate with a shorter progression-free survival and poor clinical outcome in high but not operated intermediate-risk prostate cancer patients, indicating its correlation with tumour grade (126). Similarly, LILRB1 was found in the highest grade glioma patients, and correlated with M2 macrophage markers, proliferation, migration and invasion of glioma cells, lack of response to immunotherapy and poor prognosis (118). Furthermore, blocking of LILRB1 combined with rituximab and anti-CD47 enhanced antibody-dependent cellular phagocytosis (ADCP) of chronic lymphocytic leukaemia (CLL) cells (285). *LILRB1* expression has been associated with poor AML survival, adverse prognostic impact, the inhibition of genes related to immune activation and dysfunctional CD8^+^ T cells (119). Expectedly, LILRB1 ligation by HLA-G on tumour cells induces tumour immune-evasion (206–208, 210, 211, 213, 281, 282).

LILRB2 is expressed on several types of cancers, including colon, breast, pancreas, lung, hepatocellular and prostate cancers and leukaemia (82, 126). In prostate cancer, LILRB2 together with LILRB3 and LILRB5 expression have been associated with reduced recurrence-free survival in intermediate but not high-risk patients (126). Furthermore, its overexpression in hepatocellular carcinoma, colon and NSCLC is associated with poor prognosis (82). Interestingly, in colorectal cancer it has been recently described that tumour-derived LILRB2 promotes tumour growth by increasing angiogenesis, and its blocking sensitises tumours to bevacizumab (anti-VEGF-A) treatment (163). As such, LILRB2 binding to HLA-G is associated with advanced stage and poor OS due to the increase in proliferation, migration and invasion of colorectal cancer cells (164). Moreover, in clear cell renal cell carcinoma LILRB2 increases the infiltration of macrophages, which have pro-angiogenic functions, and induces VEGF-C production (165). Additionally, LILRB2 is found on stromal macrophages, fibroblasts and plasma cells within the TME of primary breast cancer patients (162). Expression of LILRB2 on tumours correlates with higher levels of IL-10. Elevated levels of IL-10 in LILRB2^+^ breast cancer tissue positively correlates with advanced disease and lymph node metastasis, as well as reduction in tumour-infiltrating lymphocytes (TIL) (162). Moreover, LILRB2^+^ tissues in NSCLC have reduced numbers of TILs compared to LILRB2^-^ tissues (121). In addition, LILRB2 is upregulated in NSCLC patients, inducing M2 macrophage polarisation and impairing T cell function, whose inhibition reverses its immunosuppressive role (286). Moreover, *LILRB2* expression is associated with adverse prognostic impact in AML patients and lower OS (119). The ANGPTL2-LILRB2 interaction contributes to metastasis of pancreatic and lung cancers, correlating with poor survival. Oncogene mutations important in the carcinogenesis of PDA lead to the overexpression of LILRB2 and secretion of ANGPTL2 in pancreatic cancer lesions (284, 287).

LILRB3 has not yet been extensively studied with respect to tumour immune-evasion and development. LILRB3 has been found to be expressed in leukaemia and a few solid cancers, such as hepatocellular and colorectal cancers, and its expression is associated with a poor OS (119, 122, 179, 288). Perna et al., identified LILRB3 as being overexpressed on primary human AML samples and leukemic stem cells, while absent on healthy HSCs (178). Moreover, LILRB3 expression is linked to adverse prognostic impact in AML patients, with the highest LILRB3 expression found in M5 monocytic AML subtype, which correlates with worse OS. LILRB3 activates TRAF2 in AML cells, but not healthy monocytes, promoting NF-κB signalling and inhibiting anti-tumoural T cell activity (119, 288, 289). Interestingly, ectopic expression of LILRB3 on colorectal cancer cells associates with lower TILs and its high expression within the TME correlates with worse OS (179). LILRB3 as well as LILRA6 (> 90% extracellular homology) have been found to interact with cytokeratin-associated proteins on necrotic glandular epithelial cells, which may enhance tumour immune-evasion (89).

LILRB4 is expressed on a number of cancers, including AML, multiple myeloma, gastric cancer, melanoma, colorectal, pancreatic, hepatocellular, NSCLC and ovarian cancers (119, 158, 158, 185, 191–196, 290). LILRB4 has been associated with tumour immune-evasion with lower expression correlating with higher sensitivity to killing by NK cells in gastric cancer (120). LILRB4 is highly expressed on MDSCs of patients with NSCLC, correlating with poor OS due to the immunosuppressive environment and the enhanced migration, invasion and pro-angiogenic ability of NSCLC cells by binding to APOE (195, 196). In addition, LILRB4 expression on tumour-infiltrating cells and particularly MDSCs correlates with postoperative recurrence and shorter OS and relapse-free survival (290). Interestingly, its blockade prevented leukaemia metastasis and enhanced immunotherapy (185). Moreover, LILRB4 has been found on TAMs in several cancer types, and its blocking enhances the infiltration of anti-tumour immune cells due to the increased secretion of IL-1β and inducible nitric oxide synthase (184). sLILRB4 has been associated with immunosuppression and is found elevated in the sera of cancer patients (melanoma, colorectal and pancreatic), raising the possibility that it contributes to tumour escape (158). Humanised-SCID mice transplanted with several different allogenic tumour cell lines developed tumours when injected simultaneously with sLILRB4, unlike tumour cells that were injected alone (158). TILs found in these tumours were anergic with no tumour cell necrosis observed (158). T cells isolated from lymph nodes of the sLILRB4-treated mice failed to elicit T cell proliferation in a mixed lymphocyte reaction (MLR), a phenomena also observed when a MLR was conducted using human sera from cancer patients (158). The addition of a LILRB4 mAb or depletion of sLILRB4 increased T cell reactivity, demonstrating that sLILRB4 in the sera of cancer patients inhibits T cell proliferation (158). Deng et al. reported a potential mechanism for LILRB4-mediated AML progression (185). LILRB4 expression was shown to be restricted to monocytic AML cells, with ligation by APOE recruiting SHP-2 to the phosphorylated ITIM, leading to regulation of the NF-κB pathway and T cell suppression (185).

LILRB5 is also expressed in different tumours but its functions remain unclear (119, 122, 203). *LILRB5* mRNA has been detected in NK cells (203), with NK cells from hepatocellular cancer patients blood expressing higher levels of LILRB5 than those from healthy donors. The same was observed in TAMs compared to healthy tissue (122). Moreover, and opposite to other members of the family, *LILRB5* was associated with a favourable outcome in AML patients (119).

The mouse homolog PIR-B inhibits CD8^+^ T cell infiltration and promotes M2 macrophage polarisation (179). PIR-B^-/-^ MDSCs exhibit an M1-like phenotype upon entry into the periphery and result in reduced suppressive function associated with impaired Treg activity, and accelerated lung tumour growth and metastases (291). In addition, deficiency in PIR-B results in increased differentiation of AML cells, indicating that PIR-B maintains AML cell stemness and promotes leukaemia development by arresting transformed cells in an undifferentiated state (146).

### 2.6 Therapeutic potential of LILRs

Recent success in the use of immune checkpoint blockade, including pembrolizumab, nivolumab (anti-PD-1) and ipilimumab (anti-CTLA-4), has paved the way for the development of novel immune checkpoint inhibitors (292). Apart from their potential use to predict immunotherapy responses, the powerful immunomodulatory capacity of LILRs supports this family of receptors as potential therapeutic targets (119). Several immunomodulatory approaches have been proposed to target LILR family members. In particular, antibodies can exert potent immunomodulatory functions with the ability to either activate (agonistic) or block (antagonistic) the activity of the respective targets (249).

#### 2.6.1 LILR immunomodulation in infection, autoimmunity and transplantation

Targeting LILRBs could achieve allotransplantations and prevent autoimmunity. Work by Suciu-Foca and colleagues has highlighted the Ag-specific immune tolerance mediated by LILRB2^+^ LILRB4^+^ tDCs and proposed them as therapeutic targets for allotransplantation, while avoiding the side effects of indiscriminate immunosuppression (293). *Ex vivo* expansion of tDCs with T suppressor cells may enable the transfer of donor-specific tolerance to mediate transplant tolerance. Moreover, treatment with sLILRB4 has the potential to dampen over-active immunity but may lack specificity (293). In addition, treatment with recombinant human LILRB4-extracellular domain-Fc fusion-protein has been shown to induce DC tolerance, reducing the progression of the disease, while blocking it exacerbated SLE (187). Alternatively, agonistic LILRB mAbs that block immune effector functions can be used to treat autoimmune syndromes that involve exacerbated immune activation. We recently demonstrated the potential of an agonistic LILRB3 mAb to reprogram myeloid cells (177). LILRB3 mAb treatment induced tolerance *in vivo* and enabled successful engraftment of allogeneic tumour cells in a humanised mouse model. This immunosuppressive efficacy may be exploited as a therapy for transplantation and autoimmunity (177). Another study showed that GA acts as a ligand for PIR-B, LILRB2 and LILRB3 on MDSCs, whose activation promotes Th2 immunity and the release of cytokines that suppress autoimmunity (253).

LILRAs can also be used as immune modulators. In CLE, pDCs expressing LILRA4 are essential in the immunopathology of the disease. For that reason, blocking LILRA4 can improve CLE patients’ outcome. In this regard, an anti-LILRA4 (clone VIB7734) that can deplete pDCs, reduced type I IFN release and disease severity in the skin (255). In fact, this mAb was evaluated in two Phase 1 clinical trials in patients with different type I IFN-mediated autoimmune diseases, including CLE. However, the primary endpoint in the Phase 2 trial was not met. Nevertheless, a Phase 2 clinical trial in SLE was recently completed. Additionally, the same mAb has been investigated in a Phase 1 clinical trial to treat and prevent acute lung injury in patients with COVID-19 (**Table 2**).

**Table 2:** A comprehensive list of clinical trials investigating the therapeutic targeting of LILRs to date.

Therapeutic agent	Target	Format	Manufacturer	Disease indication	Monotherapy/Combination Therapy	Trial Phase	Trial Number	Completion Date
***VIB7734***	LILRA4	Humanised IgG1 mAb	Viela Bio	Dermatomyositis, Polymyositis, SS, SLE, Systemic Sclerosis	Monotherapy	1	NCT02780674	November 2017
***VIB7734***	LILRA4	Humanised IgG1 mAb	Viela Bio	Dermatomyositis, Polymyositis, SS, SLE, CLE, Systemic Sclerosis	Monotherapy	1	NCT03817424	July 2020
***VIB7734***	LILRA4	Humanised IgG1 mAb	Viela Bio	SLE	Monotherapy	2	NCT04925934	June 2023
***VIB7734***	LILRA4	Humanised IgG1 mAb	Viela Bio	Acute Lung Injury in COVID-19 patients	Monotherapy	1	NCT04526912	May 2021
***BND-22***	LILRB1	Humanised IgG4 mAb	Biond Biologics	Head and neck SCC, gastric or gastroesophageal junction adenocarcinoma and NSCLC	Monotherapy and combined therapy with pembrolizumab (anti-PD-1) or cetuximab (anti-EGFR)	1/2	NCT04717375	January 2024
***AGEN1571***	LILRB1	Humanised IgG4 mAb	Agenus	Advanced solid tumours	Monotherapy and combined therapy with balstilimab (anti-PD-1) and/or botensilimab (anti-CTLA-4)	1	NCT05377528	January 2027
***ADA-011***	LILRB1	Humanised mAb	Adanate	Advanced solid tumours	Monotherapy and combined therapy with anti-PD-L1	1	NCT05061219	December 2025
***JTX-8064***	LILRB2	Humanised IgG4 mAb	Jounce Therapeutics	Advanced solid tumours	Monotherapy and combined therapy with pimivalimab (anti-PD-1)	1/2	NCT04669899	January 2024
***ES009***	LILRB2	Humanised IgG4 mAb	Elpiscience Biopharma Australia Pty. Ltd.	Advanced solid tumours	Monotherapy	1	NCT06007482	August 2025
***CDX-585***	LILRB2 and PD-1	IgG-scFv	Celldex Therapeutics	Gastric, head and neck, ovarian, fallopian tube, bladder, colon, rectum, oesophagus, liver and pancreas cancer primary peritoneal carcinoma, NSCLC and cholangiocarcinoma	Monotherapy	1	NCT05788484	February 2026
***anti-ILT3*** ***CAR-T***	LILRB4	–	Carbiogene Therapeutics Co.Ltd Zhejiang Provincial People’s Hospital	AML M4 and M5	Monotherapy	1	NCT04803929	March 2026
***anti-ILT3*** ***STAR-T***	LILRB4	–	Peking University People’s Hospital Beijing Qingyi Taike Pharmaceutical Technology Co., Ltd	AML	Monotherapy	1	NCT05548088	August 2024
***anti-ILT3*** ***STAR-T***	LILRB4	–	Hematology and Blood Diseases Hospital	Monocytic leukaemia	Monotherapy	–	NCT05739409	August 2024
***IO-202***	LILRB4	Humanised IgG1 mAb	Immune-Onc Therapeutics California Institute for Regenerative Medicine (CIRM)	AML with monocytic differentiation and chronic myelomonocytic leukaemia	Monotherapy and combined therapy with azacitidine (chemotherapy) and azacitidien and Venetoclax (BCL-2 inhibitor)	1	NCT04372433	May 2025
***IO-202***	LILRB4	Humanised IgG1 mAb	Immune-Onc Therapeutics	Advanced solid tumours	Monotherapy and combined therapy with pembrolizumab	1	NCT05309187	April 2024
***IOS-1002***	LILRB1, LILRB2 and KIR3DL1	HLA-B57-Fc fusion protein	ImmunOs Therapeutics AG	Advanced solid tumours	Monotherapy and combined therapy with an experimental anti-PD-1	1	NCT05763004	March 2025
***IO-108***	LILRB2	Humanised IgG4 mAb	Immune-Onc Therapeutics Regeneron Pharmaceuticals	Advanced solid tumours	Monotherapy and combined therapy with pembrolizumab or cemiplimab (anti-PD-1)	1	NCT05054348	December 2024
***MK-0482***	LILRB4	Humanised IgG4 mAb	Merck Sharp & Dohme LLC	Relapsing and refractory AML and myelomonocytic leukaemia.	Monotherapy	1	NCT05038800	August 2025
***MK-0482***	LILRB4	Humanised IgG4 mAb	Merck Sharp & Dohme LLC	Triple negative breast cancer, glioblastoma, PDA, sarcomas and non-squamous NSCLC	Monotherapy and combined therapy with pembrolizumab and paclitaxel, nab-paclitaxel, gemcitabine, carboplatin, pemetrexed (chemotherapies)	1	NCT03918278	February 2025
***MK-0482***	LILRB4	Humanised IgG4 mAb	Merck Sharp & Dohme LLC	NSCLC	Combined therapy with pembrolizumab	2	NCT04165096	February 2032
***MK-4830***	LILRB2	Humanised IgG4 mAb	Merck Sharp & Dohme LCC	Ovarian, fallopian tube, stomach and pancreas cancer, head and neck SCC, NSCLC, glioblastoma multiforme, RCC, primary peritoneal carcinoma, triple negative breast cancer, mesothelioma, müllerian mixed mucinous, malignant Brenner’s, germ cell and sex cord and stromal tumours	Monotherapy and in combination with pembrolizumab, lenvatinib (tyrosine kinase inhibitor), carboplatin, pemetrexed, paclitaxel and cisplatin (chemotherapies)	1	NCT03564691	November 2025
***MK-4830***	LILRB2	Humanised IgG4 mAb	Merck Sharp & Dohme LCC	High-grade serous ovarian carcinoma	Combined therapy with pembrolizumab and standard chemotherapy	2	NCT05446870	June 2025
***NGM707***	LILRB1 and LILRB2	Humanised mAb	NGM Biopharmaceuticals, Inc Merck Sharp & Dohme LLC	NSCLC, mesothelioma, glioblastoma, RCC, PDA, head and neck SCC, cholangiocarcinoma, stomach, breast, ovaries, endometrium, cervix, colon, rectum and oesophagus cancers	Monotherapy and combined therapy with pembrolizumab	1/2	NCT04913337	July 2025
***NGM831***	LILRB4	Humanised IgG1 mAb	NGM Biopharmaceuticals, Inc Merck Sharp & Dohme LLC	NSCLC, head and neck SCC, primary peritoneal carcinoma, RCC, cholangiocarcinoma, melanoma, mesothelioma, bladder, breast, ovary, fallopian tube, endometrium, cervix, prostate, stomach, colon, pancreas rectum and oesophagus cancers	Monotherapy and combined therapy with pembrolizumab	1	NCT05215574	December 2024

AML, Acute myeloid leukaemia; CLE, Cutaneous Lupus Erythematosus; COVID-19, coronavirus 19; NSCLC, Non-small cell lung cancer; PDA, pancreatic ductal adenocarcinoma; SCC, Squamous cell carcinoma; SLE, Systemic Lupus Erythematosus; SS, Sjogren’s Syndrome; RCC, Renal cell carcinoma; -, Unknown.

#### 2.6.2 Therapeutic potential of LILRs for cancer immunotherapy

Immunomodulatory approaches targeting LILRs may also provide durable therapy for cancer. Blockade of LILRBs may simultaneously inhibit tumour progression and promote anti-tumour immune responses. Indeed, several studies have addressed this therapeutic potential, especially by targeting LILRB1, LILRB2 and LILRB4 (93, 119). Furthermore, combination with other classes of immunotherapies, such as anti-PD-1, are being investigated in the clinic (**Table 2**). Mandel et al. developed BND-22, a first-in-class LILRB1 blocking antibody to treat murine and humanised mouse tumour models by increasing the activity of NK and T cells and the phagocytic potential of macrophages (294). A Phase 1/2 clinical trial is currently evaluating the safety, tolerability and anti-tumour effect of BND-22/SAR444881 in advanced solid tumours (unresectable or metastatic disease). Another Phase 1 clinical trial is underway to examine the potency of AGEN1571, a novel LILRB1 mAb, as monotherapy or combined with anti-PD-1 or anti-CTLA-4 in advanced solid tumours. Interestingly, AGEN1571 can polarise macrophages towards a pro-inflammatory phenotype and enhances the activity of CD8^+^ T, NK and NKT cells in preclinical models (295). Additionally, patient recruitment is underway for a Phase 1 clinical trial involving another LILRB1 mAb, ADA-011, as monotherapy or combined with a PD-L1 inhibitor in advanced solid tumours. Moreover, a recent preclinical study showed that dual mAb blockade of LILRB1 and PD-1 enhances CD8^+^ T cell activation and as a result augments the cytolytic efficacy of bispecific T cell engager (BiTE) molecules (296). Similarly, Zhang and colleagues recently developed and tested the efficacy of an antagonistic LILRB1 mAb. They specifically focused on the activity on NK cells, where LILRB1 expression is significantly upregulated in cancer patients, and demonstrated that LILRB1 blockade increases the tumouricidal activity of NK cells against several types of human solid and haematological cancers in preclinical settings (297). In addition, blockade of LILRB1 and NKG2A mediated NK cell cytotoxic killing of primary human ALL and AML blasts (110).

Blockade of LILRB2 has been demonstrated to reduce cancer cell proliferation, migration and invasion of cancer cells (284). Preclinical studies in NSCLC showed that LILRB2 blockade reprograms immunosuppressive myeloid cells and promotes antitumour immunity via SHP1/2, AKT and STAT6 inhibition, suppressing granulocytic MDSCs and Treg infiltration and improving checkpoint inhibitor efficacy (160). Moreover, LILRB2 antagonism increases inflammatory macrophages by interfering with M-CSF maturation (160). Umiker and colleagues demonstrated that the blockade of LILRB2 with JTX-8064 in different tumour types reprogrammes macrophages and DCs by inhibiting HLA I ligand binding. In addition, JTX-8064 improved the efficacy of anti-PD-1 therapy (161). JTX-8064 is currently in a Phase 1/2 clinical trial as monotherapy or in combination with the PD-1 mAb pimivalimab in advanced refractory solid malignancies with potential clinical benefits (298). Moreover, in another Phase 1 clinical trial, a human LILRB2 mAb (MK-4830) is being used as monotherapy or in combination with pembrolizumab (anti-PD-1) to treat advanced solid tumours. Initial results showed 11 objective responses to the combination and 1 to the monotherapy with durable responses in heavily pre-treated patients and no dose-limiting toxicities. Furthermore, LILRB2 blockade alleviated the myeloid-suppressive compartment, improving T cell response to pembrolizumab (299). IO-108 is another LILRB2 mAb that is in a Phase 1 clinical trial to treat solid tumours (as monotherapy or in combination with pembrolizumab) with promising results reportedly due to its activation of cytotoxic T lymphocytes and APCs, and repolarisation of macrophages (300). NGM831, an antagonistic LILRB2 antibody, is being investigated as monotherapy or in combination with pembrolizumab in advanced or metastatic solid tumours in a Phase 1 clinical trial. In preclinical studies, NGM831 modulated tDCs to a more stimulatory and responsive phenotype, stimulating allogenic T cells in combination with anti-PD-1 (301). Additionally, a recent Phase 1 clinical trial is currently recruiting patients with advanced solid tumours to evaluate the safety, tolerability and clinical activity of a new human LILRB2 mAb (ES009). Previous *in vitro* and *ex vivo* studies demonstrated that blocking LILRB2 with ES009 reprograms myeloid cells to a pro-inflammatory phenotype and enhances T cell activation (302).

Furthermore, NGM707, a mAb that recognises both LILRB1 and LILRB2 is in a Phase 1/2 clinical trial as monotherapy or combined with pembrolizumab in advanced or metastatic solid tumour malignancies. Preliminary data from the Phase 1 trial showed that NGM707 was well tolerated and developed early signs of anti-tumour activity by reprogramming myeloid cells (303, 304). Similarly, a new Phase 1 clinical trial with IOS-1002, a first-in-class molecule that targets LILRB1, LILRB2 and KIR3DL1, is being investigated in patients with advanced solid tumours. It is being evaluated as monotherapy and in combination with anti-PD-1, and preclinical data have shown that it significantly increases the anti-tumourigenic responses of macrophages, T and NK cells (305). CDX-585 is a novel tetravalent IgG-scFv bispecific antibody targeting both PD-1 and LILRB2, which is undergoing Phase 1 clinical trial in advanced malignancies. In preclinical studies, CDX-585 improved T cell activation, resulted in macrophage polarisation towards M1 and enhanced anti-tumour responses in a humanised mouse model of melanoma (306).

LILRB4 has also been widely evaluated for its therapeutic potential (307). IO-202 is being investigated in relapsed/refractory myelomonocytic and monocytic AML and relapsed/refractory chronic myelomonocytic leukaemia (308). In addition, IO-202 is undergoing clinical trials as monotherapy or in combination with pembrolizumab in solid tumours (308). Similarly, Di Meo and colleagues developed a LILRB4 BiTE that showed a high efficacy in potentiating T cell cytotoxicity against multiple myeloma cells *in vitro* and *in vivo*, and prolonged survival of tumour-bearing mice (193). MK-0482 is being tested in patients with relapsed/refractory myelomonocytic and monocytic AML and relapsed/refractory chronic myelomonocytic leukaemia. However, it is important to highlight its toxicity; myositis was observed in two patients and led to death of one of them. The same antibody is being used as monotherapy or in combination with pembrolizumab in a Phase 1 clinical trial in heavily pre-treated advanced solid tumours and in a Phase 2 clinical trial in advanced NSCLC. A novel anti-LILRB4 chimeric antigen receptor (CAR) T cell therapy recently demonstrated potent elimination of human LILRB4^+^ AML cells in preclinical models with no toxicity on normal CD34^+^ hematopoietic cells (307). An early Phase 1 clinical study is evaluating the safety and efficacy of this anti-LILRB4 CAR-T cell immunotherapy in AML patients. Similarly, LILRB4 synthetic T cell antigen receptor (STAR)-T cells have been developed and are in a Phase 1 clinical trial for the treatment of relapsed/refractory AML (309) and monocytic AML. Interestingly, Huang and colleagues developed a bispecific LILRB4 x CD3 antibody for monocytic AML with promising preclinical results (310). Moreover, a T-cell engager targeting LILRB4, NGM936, to treat AML has been developed, which induces T cell cytotoxicity against LILRB4^+^ cells in preclinical studies (311). Finally, an antibody-drug conjugate has been developed from a humanised anti-LILRB4, inducing cytotoxicity against LILRB4^+^ AML cells (312).

## 3 Conclusions

LILRs are emerging as important mediators of immune homeostasis, regulating the balance between tolerance and immune activation. Increasing evidence supports LILRs’ central involvement in various human pathologies, ranging from oncology to autoimmune disorders, associated with suppressed immunity or exacerbated immune activation, respectively. Our understanding of the human LILR biology and crosstalk is limited by our understanding of their ligands, with ligands for only some LILRs identified to date. The lack of direct LILR homologues in the mouse and specific reagents have impaired the study of this important family of immune receptors. This poses a major hurdle for studying LILR biology and requires the need for the development of novel mouse models, including LILRA and/or LILRB transgenic mice and knock-in/-out mice (eg, PIR^-^ and LILR^+^), faithfully expressing these receptors. As such, more recent engineering advances in generating humanised mice have begun to allow the study of this complex receptor family in a more ‘physiological’ context and will undoubtedly continue to support the study of these receptors and other elusive immune receptors (177, 218, 313, 314). These models could allow us to further examine the ligand profiles, functions and therapeutic potential of the LILR family members.

In regard to their therapeutic potential, the high homology among certain LILRs must be taken into consideration. Without this, targeting the inhibitory LILRs could simultaneously mediate LILRA activation or inhibition, which may interfere with the desired outcome. The recent preclinical and clinical evidence propose LILRBs as ideal targets for immunotherapies against various pathologies including cancer. Most exciting is the emerging dual role of LILRBs in promoting carcinogenesis and immune-evasion, which propose the development of novel and highly potent immunotherapies for reducing tumour burden and immunosuppression. As such, it is anticipated that novel LILR-targeting modalities currently in clinical trials will soon make their way into the clinic.

## Author contributions

SR-G: Writing – original draft, Writing – review & editing. CB: Writing – original draft. CP: Writing – original draft, Writing – review & editing. MY: Writing – original draft. BF: Writing – review & editing. MSC: Funding acquisition, Supervision, Writing – review & editing. AR: Conceptualization, Funding acquisition, Supervision, Writing – original draft, Writing – review & editing.
